# A Supramolecular, Triple Negative Breast Cancer‐Targeting Avidin‐Photosensitizer

**DOI:** 10.1002/mabi.202400610

**Published:** 2025-03-25

**Authors:** Bingjie Gao, Charlotte Schäfers, Seah Ling Kuan, Tanja Weil

**Affiliations:** ^1^ Department of Synthesis of Macromolecules Max Planck Institute for Polymer Research 55128 Mainz Germany; ^2^ Johannes Gutenberg University Duesbergweg 10–14 55128 Mainz Germany

**Keywords:** photodynamic therapy, supramolecular protein‐drug conjugates, targeted delivery, triple‐negative breast cancer

## Abstract

The potential of photodynamic therapy (PDT) in combination with chemotherapy to improve treatment outcomes for triple‐negative breast cancer (TNBC), for which no targeted therapy is available, is the subject of considerable investigation. In PDT, photosensitizers (PSs) are frequently administered directly but do not selectively target cancer cells. To address the delivery of a PS to TNBC and enhance cellular uptake, the Ru‐NH_2_‐modified avidin bioconjugate (^Ru^Avi) via Tyr‐specific modification using the Mannich reaction is prepared. The ^Ru^Avi is further assembled with the cinnamoyl peptide‐F(D)LF(D)LFK‐NH_2_ (FK), which binds to formyl peptide receptor 1, overexpressed in TNBC. Notably, the modified Avi still possesses the ability to efficiently bind biotin for the assembly of up to four copies of the FK peptides. The resultant FK_4_‐^Ru^Avi exhibited an IC_50_ value of 0.36 ± 0.08 µM, which is ≈3.5‐fold lower than that of ^Ru^Avi (1.25 ± 0.09 µM), upon irradiation in the triple‐negative MDA‐MB‐231 breast cancer cells. FK_4_‐^Ru^Avi also shows efficient uptake in MDA‐MB‐231 tumor spheroids and exhibited significant toxicity after irradiation compared to the control ^Ru^Avi. The presented strategy has the potential to improve the efficacy of targeted PDT to meet the high demand for targeted therapies to treat TNBC, such as targeted adjuvant treatment after breast cancer surgery.

## Graphical abstract

This study investigates the targeted delivery of photosensitizers in triple‐negative breast cancer (TNBC) using a Ru‐NH_2_‐modified avidin bioconjugate (^Ru^Avi). ^Ru^Avi binds to the cinnamoyl peptide, F(D)LF(D)LFK‐NH_2_ (FK), targeting the formyl peptide receptor 1, which is overexpressed in TNBC. The resulting FK_4_‐^Ru^Avi shows enhanced internalization and significant phototoxicity in TNBC cells and spheroids, which is promising for targeted PDT.

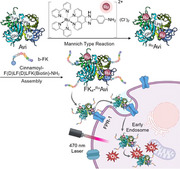

## 1 Introduction

Breast cancer is the second most common cancer worldwide.^[^ ^1^ ^]^ Triple‐negative breast cancer (TNBC) is a subtype of breast cancer that accounts for ≈10%–20% of breast cancer diagnoses.^[^ ^2^ ^]^ TNBC is characterized by the absence of estrogen receptor (ER), progesterone receptor (PR), and human epidermal growth factor receptor 2 (HER2) expression and is associated with high distant metastatic potential, aggressive behavior, and short overall survival.^[^ ^3^ ^]^ Although the primary treatment for TNBC is chemotherapy, other treatment options include surgery, radiotherapy, and immunotherapy.^[^ ^4^ ^]^ However, due to its more aggressive characteristics and poorer prognosis than other breast cancer subtypes, there is a need to develop specific, effective treatment options for TNBC patients.^[^ ^5^ ^]^

Photodynamic therapy (PDT) has been investigated in combination with chemotherapy to offer better treatments for TNBC.^[^ ^6^ ^]^ To reduce the risk of recurrence, PDT is also being investigated to remove microscopic residual tumors in the breast and surrounding tissue, post‐surgery.^[^ ^7^ ^]^ In PDT, a photosensitizer (PS) is excited by light to generate reactive oxygen species (ROS) to damage cells and tissues.^[^ ^8^ ^]^ For example, 5‐aminolevulinic acid (ALA) has been approved by the FDA as a photosensitizing prodrug.^[^ ^9^ ^]^ Recently, 5‐ALA monotherapy was shown to significantly reduce the viability of MDA‐MB‐231 breast cancer cells after irradiation at various doses.^[^ ^6b^ ^]^ Although ALA can be administered locally or systemically and has rapid clearance, like many PS used in PDT, it suffers from indiscriminate biodistribution. Consequently, its application is limited due to the phototoxicity of PS in healthy cells. Therefore, a PS attached to a soluble and biodegradable transporter, for targeted delivery to breast cancer cells, would be valuable to improve treatment efficacy and overcome some of the current limitations.^[^ ^10^ ^]^

Formyl peptide receptor 1 (FPR‐1) is a G protein‐coupled membrane protein receptor (GPCR) that is expressed in several types of cancer, including TNBC.^[^ ^11^ ^]^ In addition to its primary role in the immune response, the FPR‐1 receptor is involved in several stages of cancer progression, including cancer cell migration and invasion.^[^ ^12^ ^]^ The cinnamoyl peptide F(D)LF(D)LFK‐NH_2_ (FK) has been identified as an antagonist of FPR‐1, exhibiting a targeting effect upon binding to the FPR‐1 receptor.^[^ ^13^ ^]^ Nevertheless, there is limited investigation of delivery systems with FRP‐1‐mediated uptake for cancer therapy. Therefore, this could be an attractive avenue for further research, with the potential to enhance the uptake of PSs into TNBC cells. A transporter carrying multiple copies of FPR‐1 targeting groups and a PS could potentially combine efficient phototoxicity and selectivity for cancer cells.

In the past, proteins have been widely employed as drug transporters due to their biocompatibility and biodegradability.^[^ ^14^ ^]^ For example, the protein avidin, derived from egg whites, has been employed as a platform for the assembly of supramolecular bioconjugates.^[^ ^13a^ ^]^,^[^ ^15^ ^]^ Avidin is a tetrameric protein that exhibits high affinity and specificity for biotin (vitamin H) molecules, with a dissociation constant (K_d_) of ≈10^−15^ m.^[^ ^16^ ^]^ The biotinylated molecules can be assembled onto avidin, and the simultaneous binding of multiple biotinylated molecules has been achieved previously. Given its robust, rapid, and selective interaction with biotin, avidin has been extensively utilized in a multitude of biological applications, including molecular biology assays, affinity chromatography, microscopy, and drug delivery.^[^ ^13a^ ^]^,^[^ ^15^ ^]^,^[^ ^17^ ^]^ It is noteworthy that avidin has been employed for the combination of multiple copies of targeting peptides with therapeutic cargo, including enzymes and drug molecules, which enhanced uptake into cells.^[^ ^16c^ ^]^,^[^ ^17a^ ^]^,^[^ ^18^ ^]^ The method offers advantages over the synthetic approach in terms of ease of preparation and purification. However, the intrinsic property of avidin, which has only four binding pockets, restricts the number of biotinylated entities that can be introduced.

**Scheme 1:**
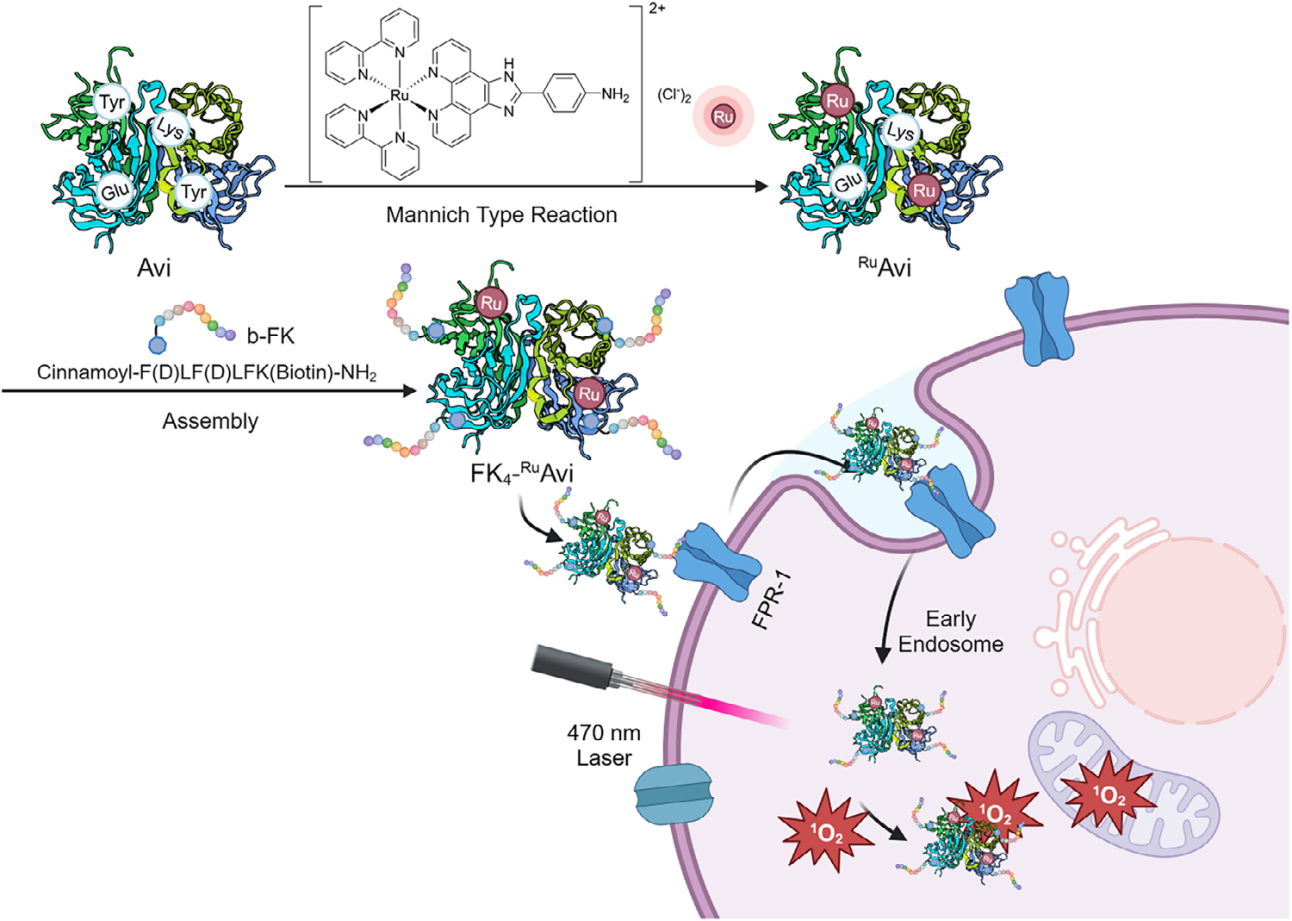
Design showing the preparation of the avidin‐PS (^Ru^Avi) via tyrosine modification and subsequent controlled assembly with FPR‐1 targeting b‐FK peptide for PDT applications to address TNBC.

In this study, we present the synthesis of a multifunctional avidin transporter that delivers a ruthenium (II) polypyridyl complex (Ru‐NH_2_) PSs into TNBC cells (**Scheme** **1**). The optimal avidin transporter has been prepared by the assembly of four biotinylated FK (b‐FK) peptides and two Ru‐NH_2_‐PSs, through the functionalization of surface‐exposed tyrosine residues in avidin. Tyrosine residues can undergo a Mannich‐type reaction, which is a three‐component condensation reaction involving amines, formaldehyde, and carbonyl‐containing reagents.^[^ ^19^ ^]^ Since each avidin monomer possesses only one tyrosine, this enables more controlled attachment of Ru‐NH_2_‐PSs and homogenous product, as compared to using lysine modification which can afford higher loading but gives a broader statistical distribution.^[^ ^20^ ^]^ The resulting transporter, FK_4_‐^Ru^Avi exhibits an enhanced FPR‐1‐mediated internalization in the TNBC MDA‐MB‐231 cells that overexpressed FPR‐1, as well as notable phototoxicity in both 2D cancer cells and 3D tumor spheroids. The multifunctional supramolecular protein conjugates reported here provide new avenues for the design of targeted PS‐nanoplatform for photodynamic applications to address existing challenges associated with TNBC.

## 2 Results and Discussion

### 2.1 Preparation and Characterization of ^Ru^Avi

We have designed a TNBC cell surface receptor‐targeting, multifunctional avidin‐PS derivative with optimized cellular uptake through the assembly of up to four b‐FK peptides. For the attachment of the PS, low abundant amino acids were identified in order to minimize structure perturbations and retain the biotin‐binding of the avidin transporter.^[^ ^21^ ^]^ Tyrosine residues occur in low abundance on the avidin surface, i.e., only one per monomer, and their functionalization should not affect the tetramer's structural integrity and biotin‐binding ability.^[^ ^19^, ^21^, ^22^ ^]^ Inorganic PS such as Ru complexes are widely used as PSs due to their favorable photophysical properties,^[^ ^23^ ^]^ as well as stability and low dark toxicity in comparison to organic PSs. Ru‐NH_2_ operates primarily via a type II photosensitization mechanism, with a quantum yield of 4.8%, which is typical for Ru (II) polypyridyl complexes.^[^ ^19a^ ^]^ Thus for our proof‐of‐concept study, we used a Ru‐NH_2_, as a representative inorganic PS (Figure S1, Supporting Information).^[^ ^19a^ ^,^ ^24^ ^]^

**Figure 1:**
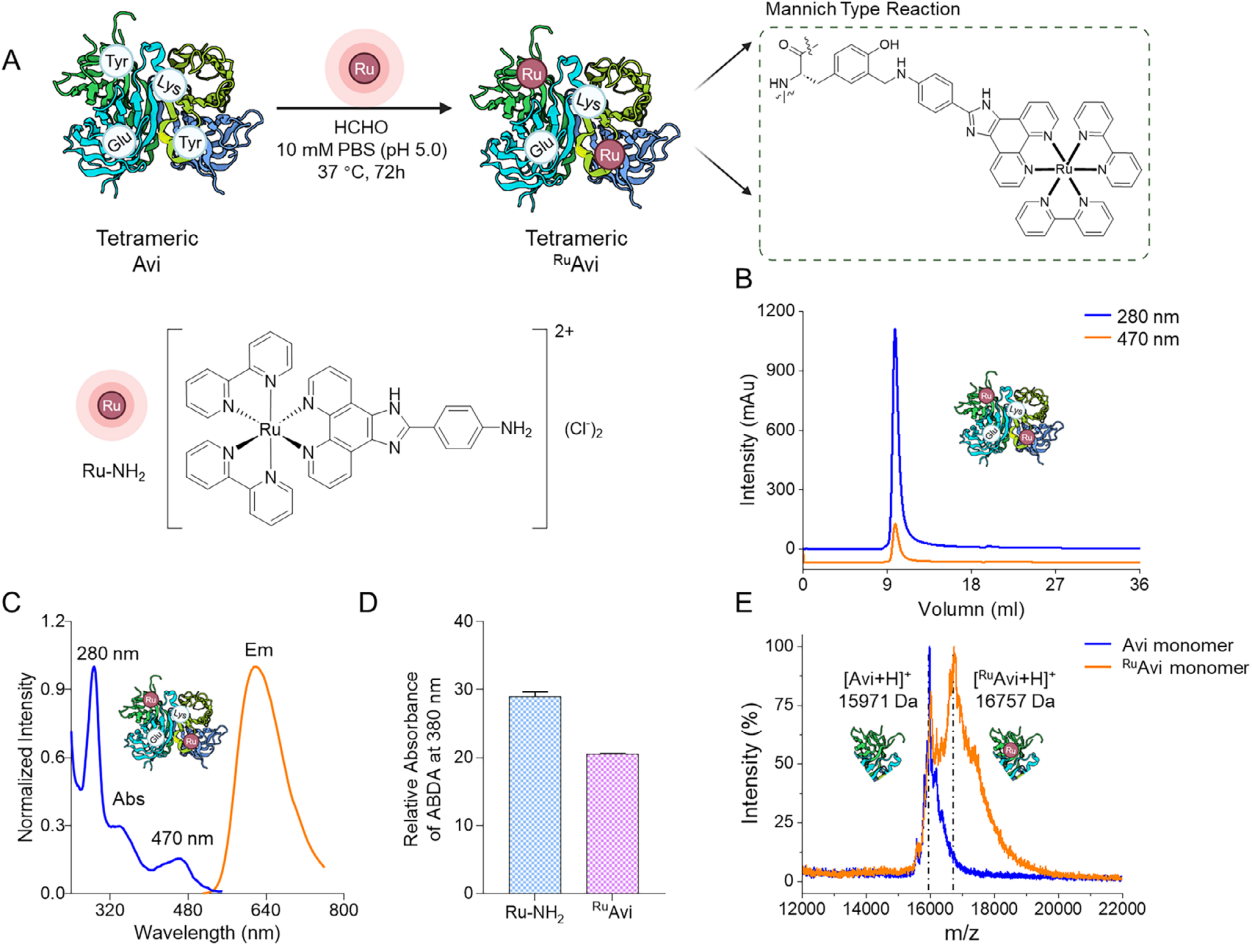
A) Synthesis of ^Ru^Avi and the chemical structure of Ru‐NH_2_. B) Size exclusion chromatography characterization of ^Ru^Avi tetramer under absorption at 280 nm (blue) and 470 nm (orange) within an ÄKTA pure™ protein purification system. C) Normalized UV–vis absorption (blue) and fluorescence emission (orange) spectra of ^Ru^Avi tetramer after excitation with 470 nm. D) Singlet oxygen (^1^O_2_) production of ^Ru^Avi and Ru‐NH_2_, measured by the photobleaching of the characteristic absorption peak at 380 nm of ABDA after irradiation with 470 nm LED arrays (20 mA, 10 min). The absorption intensity was plotted relative to the value before irradiation. The data is shown as mean ± SEM, n = 3. E) MALDI‐ToF MS characterization comparing monomeric ^Ru^Avi (orange) and native Avi (blue). MALDI‐ToF MS for 10–40 kDa is given in Figure S6 (Supporting Information).

Tyrosine was functionalized first by a three‐component Mannich‐type coupling reaction with an amine in a phenanthroline‐based ligand of Ru‐NH_2_.^[^ ^19^ ^]^ A 15 µm Avi solution was mixed with 750 µm formaldehyde and 750 µm Ru‐NH_2_ at 37 °C for 72 h (**Figure** **1**A). The mixture was subsequently purified by centrifugal ultrafiltration and characterized by size exclusion chromatography in an ÄKTA pure^TM^ protein purification system (Figure 1B; Figures S2,S3, Supporting Information). When eluting 10 mL of 50 mm phosphate buffer (PB, pH 7.4), only one fraction with absorption at both 280 and 470 nm appeared, with no detectable free Ru‐NH_2_ and the retention volume indicated a tetrameric ^Ru^Avi conjugate, without the formation of aggregates.

We further characterized tetrameric ^Ru^Avi by UV–vis spectroscopy. The spectrum showed two absorption peaks, a protein‐related absorption located at 280 nm and a Ru‐NH_2_‐related absorption located at 470 nm, with a corresponding fluorescence emission at 640 nm (λ_ex_ = 470 nm, Figure 1C). The degree of modification was determined to be 57%, accounting for 2.3 Ru‐NH_2_ per tetrameric ^Ru^Avi by plotting a calibration curve using the absorption at 470 nm of Ru‐NH_2_ standard solutions varying from 0.001 to 1 mg mL^−1^ as references (Figure S4, Supporting Information). To investigate the ability of ^Ru^Avi to generate singlet oxygen (^1^O_2_), the ^1^O_2_ sensor 9,10‐anthracenediyl‐bis(methylene)dimalonic acid (ABDA) was employed (Figure 1D; Figure S5, Supporting Information). ABDA undergoes conversion to an endoperoxide in the presence of ^1^O_2_, resulting in a decrease in the absorption of ABDA. ^Ru^Avi and the control compound Ru‐NH_2_ were mixed separately with 10 mm ABDA in a 50 mm PB buffer and subsequently exposed to irradiation using a 470 nm LED array for 10 mins. We observed a comparable decrease in absorption in the ABDA assay with both ^Ru^Avi and Ru‐NH_2_ (21% and 29%, respectively). This suggests that the attachment of the Ru PS to the avidin platform does not have a significant impact on the production of ^1^O_2_. In the Matrix‐Assisted Desorption/Ionization‐Time of Flight Mass Spectrometry (MALDI‐ToF MS), Avi is detected in the monomeric form due to the ionization process. From the MALDI‐ToF MS, we determined the mean molecular weight (Mw) of the ^Ru^Avi monomer over two batches that were prepared (Figure S6, Supporting Information). Two peaks were observed in the protein sample after the Mannich reaction and purification. The peak with a Mw of 15971 ± 3 Da corresponded to an unmodified avidin monomer whereas the new peak appearing at an Mw of 16757 ± 24 Da corresponded to an avidin monomer modified with Ru‐NH_2_, with an increase of ≈786 ± 27 Da (Figure 1E). This is slightly higher than the calculated Mw of 736 and the deviation is most likely due to the broadness and low resolution of the MALDI‐ToF MS protein peak.^[^ ^25^ ^]^ The observation of the two peaks is consistent with the results from the UV–vis absorption measurement.

### 2.2 In Situ Supramolecular Assembly of b‐FK onto ^Ru^Avi Platform and Internalization into TNBC Cells

**Figure 2:**
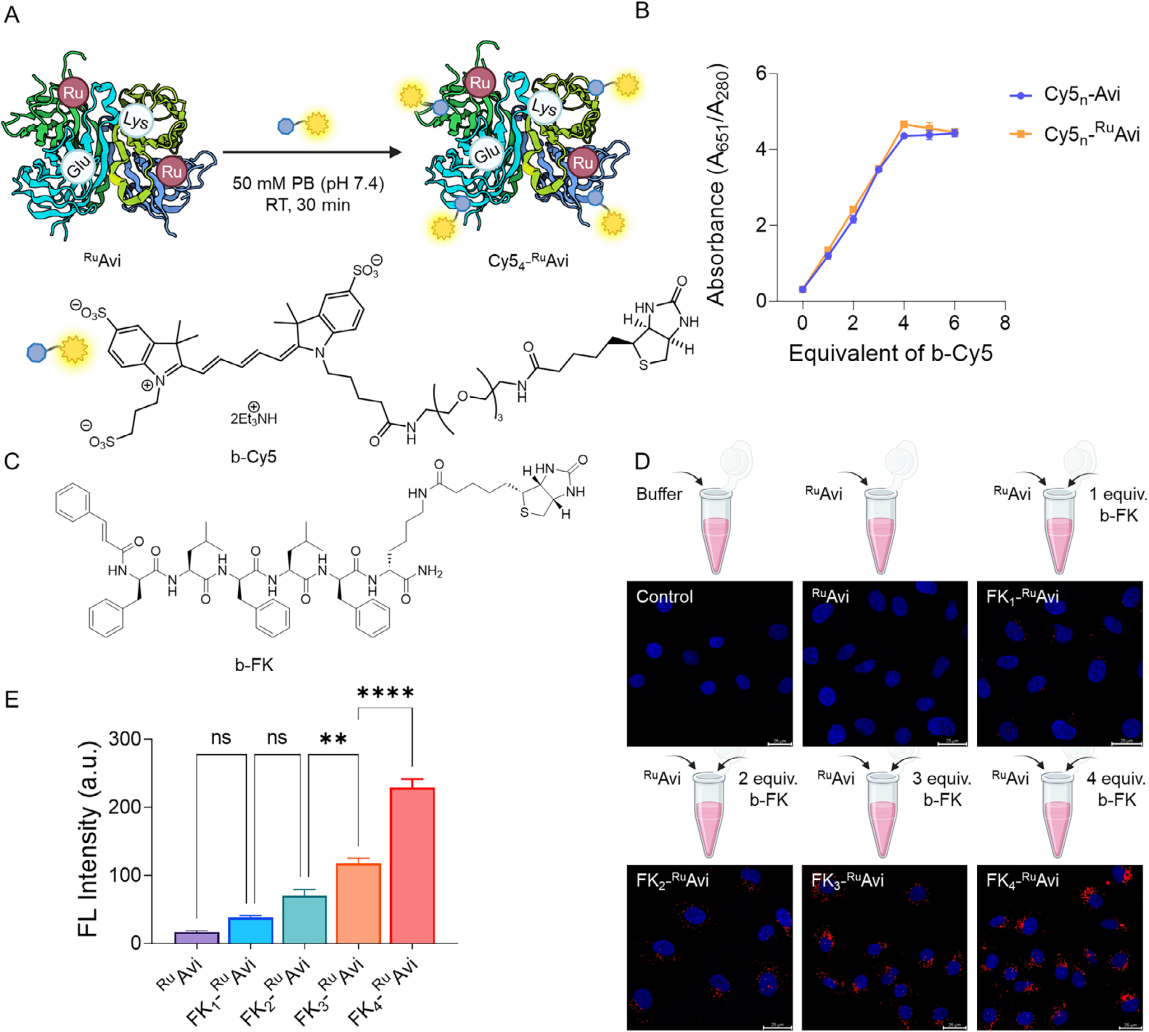
A) Scheme for assessing the availability of the biotin‐binding pockets of ^Ru^Avi through assembly with b‐Cy5 to produce Cy5_n_‐^Ru^Avi (*n* = 0–6). B) Linear plot based on the specific absorption ratios between b‐Cy5 (651 nm) and protein (280 nm) to determine stoichiometry required to saturate biotin‐binding pockets on ^Ru^Avi and Avi. C) Chemical structure of b‐FK. D) Scheme of the in situ combinatorial screening for the optimal FK_n_‐^Ru^Avi transporter, where confocal laser scanning microscopy (CLSM) showed the intracellular internalization of the transporter formed by the assembly of different equiv of b‐FK with ^Ru^Avi varying from 0 to 4 and confirmation of FK peptide mediating uptake in MDA‐MB‐231 cells (scale bar 25 µm; red channel, Ru‐NH_2_; blue channel, Nucblue). E) Quantification of internalization into MDA‐MB‐231 cell for FK_n_‐^Ru^Avi (*n* = 0–4) by detecting fluorescence intensity after cell lysis (Ex 470 nm, Em 640 nm). The data is shown as mean ± SEM, n = 5. Statistical significance was analyzed by one‐way ANOVA, ^*^ *p* < 0.05, ^**^ *p* < 0.01, ^***^ *p* < 0.001, and ^****^ *p* < 0.0001.

Next, we determined the effects of the number of FK peptides assembled onto ^Ru^Avi on internalization into TNBC cells using a combinatorial approach. Before assembly with the targeting peptides, we first assessed the availability of all four biotin‐binding pockets in ^Ru^Avi (**Figure** **2**A). Therefore, binding assays were performed with the fluorescent conjugate biotin‐PEG_3_‐Cyanine5 (b‐Cy5) as a detectable model system. 0–6 equivalents (equiv) of b‐Cy5 were used to assemble ^Ru^Avi bioconjugates with increasing numbers of Cy5‐chromophores. Excess and unbound b‐Cy5 was removed by centrifugal ultrafiltration, and the absorption spectra were acquired and processed using its characteristic absorption at 651 nm (representing b‐Cy5) and 280 nm (representing protein) to obtain the ratio A_651_/A_280_ (Figure 2B). The A_651_/A_280_ increased as b‐Cy5 increased between 0 and 4 equiv, indicating that the binding pockets of Avi and ^Ru^Avi were still not saturated. When b‐Cy5 was increased from 4 to 6 equiv, the A_651_/A_280_ remained constant, indicating that the binding pockets of ^Ru^Avi were saturated with 4 equiv of b‐Cy5. Thus, each binding pocket of ^Ru^Avi required 1 equiv of the biotinylated entity.

Based on this, we next prepared FK_n_‐^Ru^Avi in situ to determine the optimum number of FK peptides required for internalization into TNBC. FK peptides can target FPR‐1 involved in innate immunity, inflammation, and cancer, and have been reported to act in the progression of various tumor histotypes encompassing gastric, lung, breast, and cervical cancers.^[^ ^11^ ^]^ FK peptide which was biotinylated at the *C*‐terminus and purchased from AmbioPharm with 95% purity (Figure 2C) was used. 1 to 4 equiv of b‐FK peptides were used for in situ assembly with ^Ru^Avi through stoichiometric control to afford FK_n_‐^Ru^Avi (*n* = 0–4, corresponding to the number of equiv of FK added per tetrameric Avi). These assemblies were used for combinatorial screening for internalization into MDA‐MB‐231, a triple‐negative breast cancer cell line. The MDA‐MB‐231 cell line is known to have a strong proliferative capacity associated with the activation of overexpressed FPR‐1 by Annexin A1 and was therefore selected as a model cell line for in vitro experiments.^[^ ^11b^ ^]^,^[^ ^12a^ ^]^ First, the cellular internalization of FK_n_‐^Ru^Avi (*n* = 0–4) was investigated using confocal laser scanning microscopy (CLSM). The negative control ^Ru^Avi with no b‐FK assembled, showed very weak internalization after 24 h incubation (Figure 2D). With an increasing number of b‐FK, the internalization of 1 µm FK_n_‐^Ru^Avi (*n* = 0–4) became progressively more pronounced, with the optimal uptake of the FK_n_‐^Ru^Avi transporter occurring when ^Ru^Avi was saturated with four equiv of b‐FK. Quantitative analysis of FK_n_‐^Ru^Avi (*n* = 0–4) internalization in MDA‐MB‐231 cells was conducted by measuring the fluorescence intensity after cell lysis, revealing a consistent result, with gradual and significant internalization as the number of equiv of b‐FK used for assembly was increased (Figure 2E). Interestingly, compared with FK_1_‐^Ru^Avi, the increase in the degree of internalization of FK_2_‐^Ru^Avi was 2‐fold but not statistically significant, while that of FK_3_‐^Ru^Avi and FK_4_‐^Ru^Avi were statistically significant and more pronounced, with 3‐ and 6‐fold increase, respectively. This observation was most likely due to the additive effects of the multiple copies of target groups, with FK_4_‐^Ru^Avi showing the highest degree of internalization into MDA‐MB‐231 cells. FK_4_‐^Ru^Avi is selected as the optimal transporter and used for subsequent characterization and studies.

### 2.3 Characterization of FK_4_‐^Ru^Avi and In Vitro Investigation

**Figure 3:**
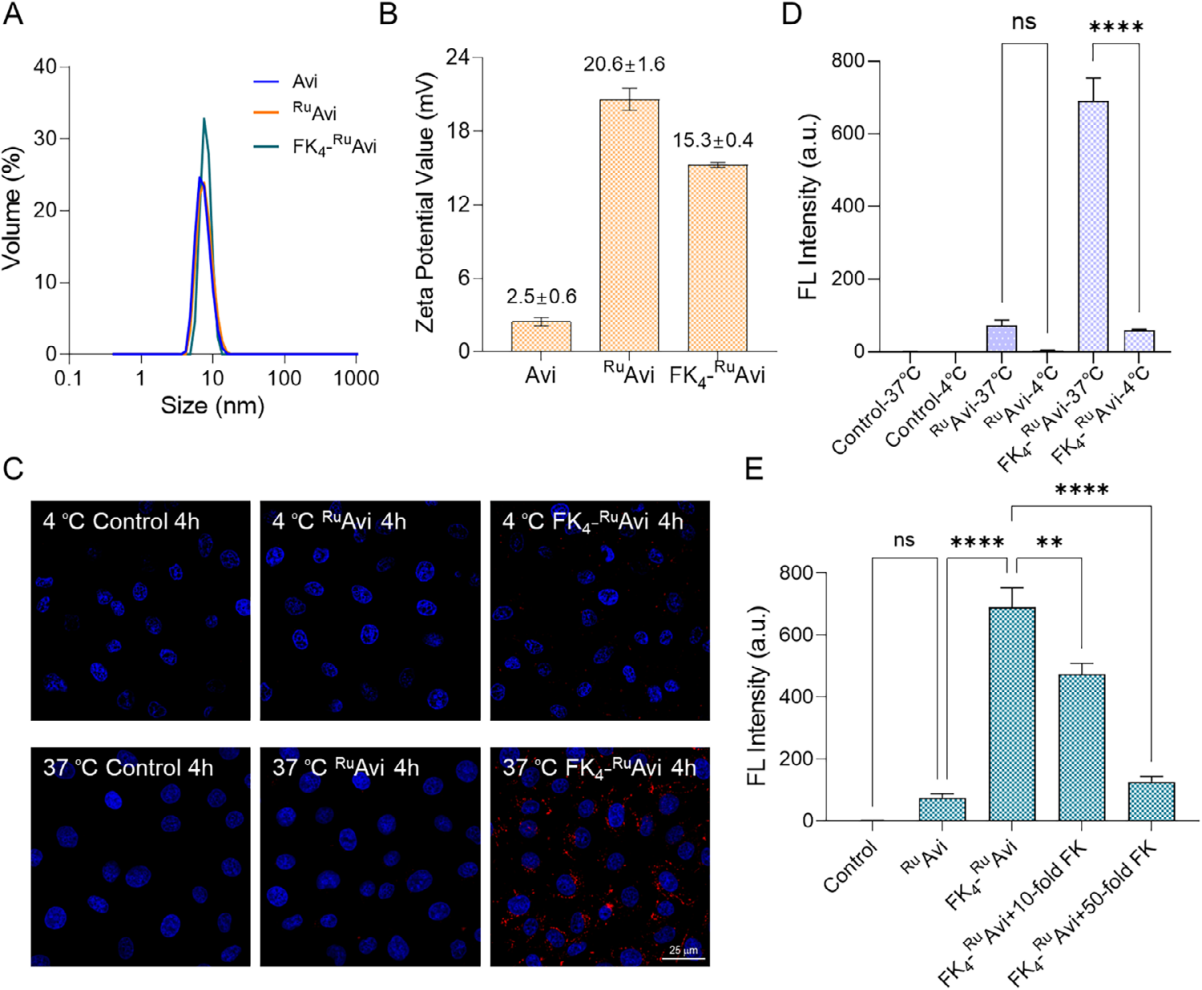
A) Size determination by DLS of 1 mg mL^−1^ FK_4_‐^Ru^Avi, ^Ru^Avi, and Avi in 10 mm PBS (pH 7.4). B) Zeta potential values of 0.2 mg mL^−1^ FK_4_‐^Ru^Avi, ^Ru^Avi, and Avi in 1 mm KCl. C) Confocal laser scanning micrographs showing the intracellular internalization of FK_4_‐^Ru^Avi and ^Ru^Avi at 4 and 37 °C for 4 h to demonstrate their temperature‐dependent internalization (scale bar 25 µm; red channel, Ru‐NH_2_; blue channel, Nucblue). D, E) Quantification of mean fluorescence intensity using ImageJ. Statistical analysis was performed using one‐way ANOVA to assess the significance of differences in cellular uptake between the samples, ^*^ *p* < 0.05, ^**^ *p* < 0.01, ^***^ *p* < 0.001, and ^****^ *p* < 0.0001. D) Incubation at 4 and 37 °C for 4 h, E) with or without excess b‐FK treatment. B, D, E) Data is shown as mean ± SEM, *n* = 3.

The average hydrodynamic diameter of the optimized FK_4_‐^Ru^Avi was determined by dynamic light scattering (DLS) to be 8.0 ± 0.5 nm, which did not show a significant increase compared to 7.4 ± 0.5 nm for ^Ru^Avi and 7.1 ± 0.7 nm for Avi (**Figure** **3**A). This is expected as the FK peptide is a short sequence and will not contribute to a significant increase in size. The zeta potential of FK_4_‐^Ru^Avi was determined to be 15.3 ± 0.4 mV, compared to 20.6 ± 1.6 and 2.5 ± 0.6 mV for ^Ru^Avi and Avi (Figure 3B and Table S1, Supporting Information). The increase in the zeta potential value of FK_4_‐^Ru^Avi and ^Ru^Avi compared to Avi is presumably due to the positively charged ruthenium complex. Furthermore, the stability of FK_4_‐^Ru^Avi was verified through sodium dodecyl sulfate‐polyacrylamide gel electrophoresis (SDS‐PAGE) (Figure S7, Supporting Information). FK_4_‐^Ru^Avi, stored at 4 °C for 1 month post‐assembly, were subjected to electrophoretic analyses, which showed that it did not disassemble to the monomer or small fragments.

Since receptor‐mediated endocytosis is a temperature‐dependent process, we treated MDA‐MB‐231 cells with FK_4_‐^Ru^Avi at 4 and 37 °C for 4 h and quantified the uptake using confocal microscopy (Figure 3C; Figure S8, Supporting Information).^[^ ^26^ ^]^ As expected, there is a temperature‐dependent internalization of the FK_4_‐^Ru^Avi complex, with a 12‐fold decrease in internalization at 4 °C compared to 37 °C (Figure 3D; Figure S9, Supporting Information). ^Ru^Avi was used as a negative control and showed little difference in uptake at the two different temperatures. To further prove the receptor‐mediated uptake, we saturated the cell surface FPR‐1 receptor with an excess of b‐FK (Figure S10, Supporting Information). With a 10‐fold excess of b‐FK, the internalization efficiency of FK_4_‐^Ru^Avi was reduced to 69% compared to the control with no b‐FK added. With a 50‐fold excess, the internalization was reduced by 82% (Figure 3E; Figure S11, Supporting Information). Taken together, these results support the receptor‐mediated uptake of FK_4_‐^Ru^Avi.

**Figure 4:**
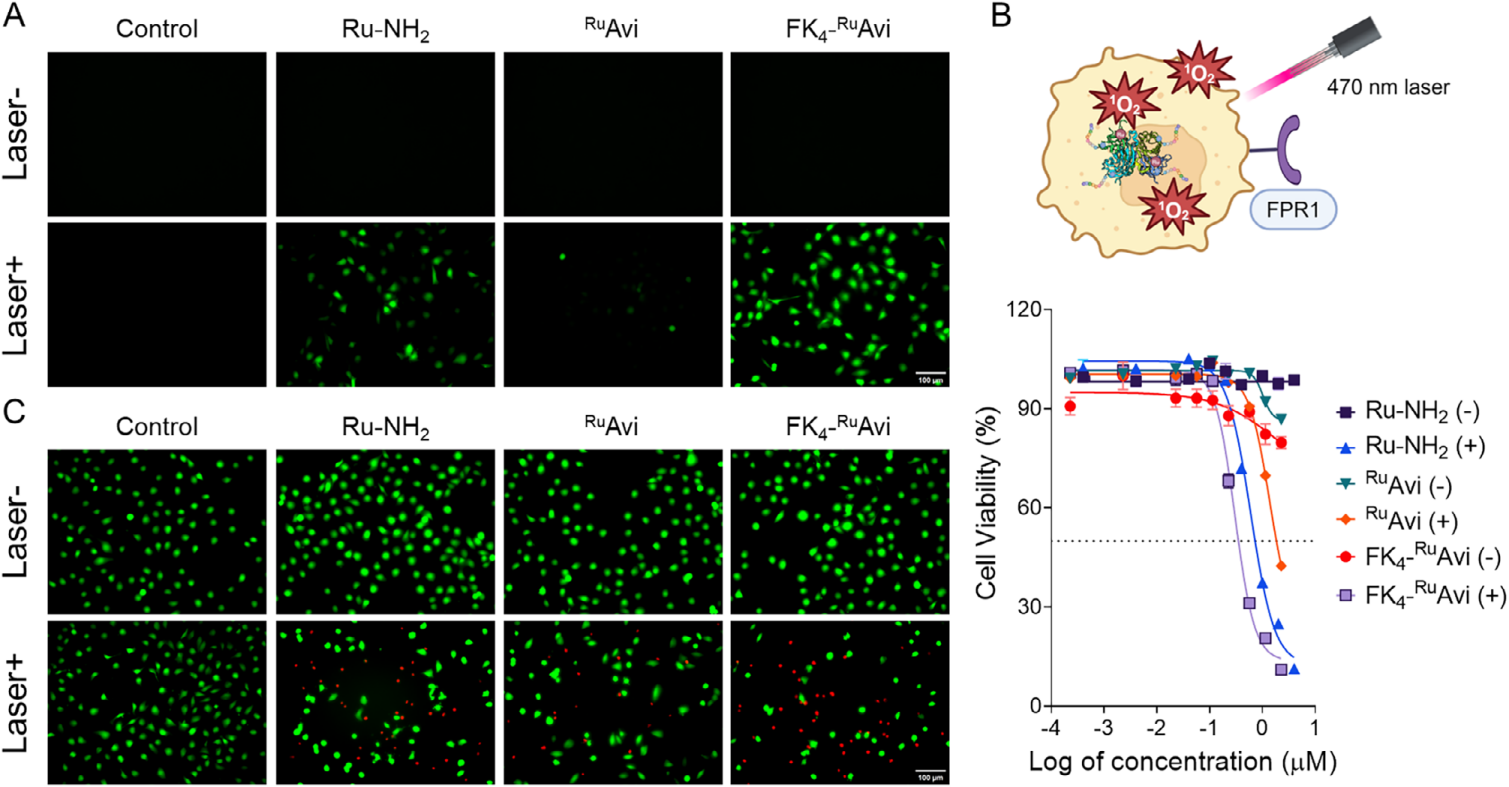
A) Intracellular ROS production detection in MDA‐MB‐231 cells by sequentially treating the cells with 2 µm Ru‐NH_2_, 0.5 µm ^Ru^Avi, 0.5 µm FK_4_‐^Ru^Avi for 24 h and stained with 10 µM DCFH‐DA for 30 min with or without laser irradiation (470 nm, 15 mA, 5 min). Scale bar 100 µm; Ex 470/40 nm, Em 525/50 nm. B) Cell viability of MDA‐MB‐231 treated with Ru‐NH_2_, ^Ru^Avi, and FK_4_‐^Ru^Avi for 48 h, with or without laser irradiation (470 nm, 15 mA, 5 min), with n = 5. C) Calcein‐AM/PI co‐staining of MDA‐MB‐231 cells after treatment with 2 µm Ru‐NH_2_, 0.5 µm ^Ru^Avi, 0.5 µm FK_4_‐^Ru^Avi for 24 h, followed with or without laser irradiation (470 nm, 15 mA, 5 min). Scale bar 100 µm; green channel, Calcein‐AM, 2 µm, Ex 470/40 nm, Em 525/50 nm; red channel, PI, 4.5 µm, Ex 545/25, Em 605/70.

For effective PDT treatment, high levels of ROS generation within cancer cells are essential. Therefore, we investigated the phototoxicity of FK_4_‐^Ru^Avi upon treatment with irradiation. First, the ROS generation by irradiation of FK_4_‐^Ru^Avi was evaluated using a 2′,7′‐dichlorofluorescein diacetate (DCFH‐DA) staining probe (**Figure** **4**A). For the untreated groups, there was almost no ROS generated, regardless of whether laser irradiation was applied or not. The experimental groups of Ru‐NH_2_, ^Ru^Avi, and FK_4_‐^Ru^Avi also produced negligible ROS without irradiation, while after laser exposure, the diffused small molecule Ru‐NH_2_ exhibited a high ROS signal. Compared with ^Ru^Avi, the assembled FK_4_‐^Ru^Avi also showed enhanced ROS generation, similar to that of Ru‐NH_2_.

The cell viability of MDA‐MB‐231 upon the treatment of FK_4_‐^Ru^Avi was then assessed using 3‐(4,5‐dimethylthiazol‐2‐yl)‐2,5‐diphenyltetrazolium bromide (MTT) assay (Figure 4B; Figures S12,S13, Supporting Information). MDA‐MB‐231 cells incubated with Ru‐NH_2_, ^Ru^Avi, and FK_4_‐^Ru^Avi without irradiation showed little cytotoxicity up to 2.3 µm (based on Ru concentration, 1 µm based on protein complex). Notably, a concentration‐dependent cytotoxicity was observed after light irradiation. The IC_50_ value was plotted based on the Ru‐NH_2_ labeling rate on ^Ru^Avi of 2.3 Ru‐NH_2_ per Avi in terms of Ru‐NH_2_ concentration. Compared to the IC_50_ value of the free small molecule Ru‐NH_2_ at 0.58 ± 0.01 µm, the IC_50_ of FK_4_‐^Ru^Avi under laser irradiation was slightly lower at 0.36 ± 0.08 µm, while the IC_50_ of ^Ru^Avi was significantly higher at 1.25 ± 0.09 µm (Table S2, Supporting Information). Furthermore, Calcein‐AM/PI (Propidium Iodide) co‐staining was performed to visualize live and dead MDA‐MB‐231 cells (Figure 4C). Without irradiation, all groups displayed a high proportion of live cells, emitting green fluorescence as stained by Calcein‐AM. Upon light irradiation, the untreated group continued to show a high proportion of live cells with bright green fluorescence. In contrast, cells pretreated with Ru‐NH_2_ and FK_4_‐^Ru^Avi showed a smaller proportion of surviving cells (green fluorescence) and a higher proportion of dead cells, indicated by the red fluorescence from PI staining. When treated with ^Ru^Avi, the proportion of surviving cells (green fluorescence) was higher than that of dead cells (red fluorescence), showing less cytotoxic effect.

### 2.4 Effect of FK_4_‐^Ru^Avi on 3D MDA‐MB‐231 Model

**Figure 5:**
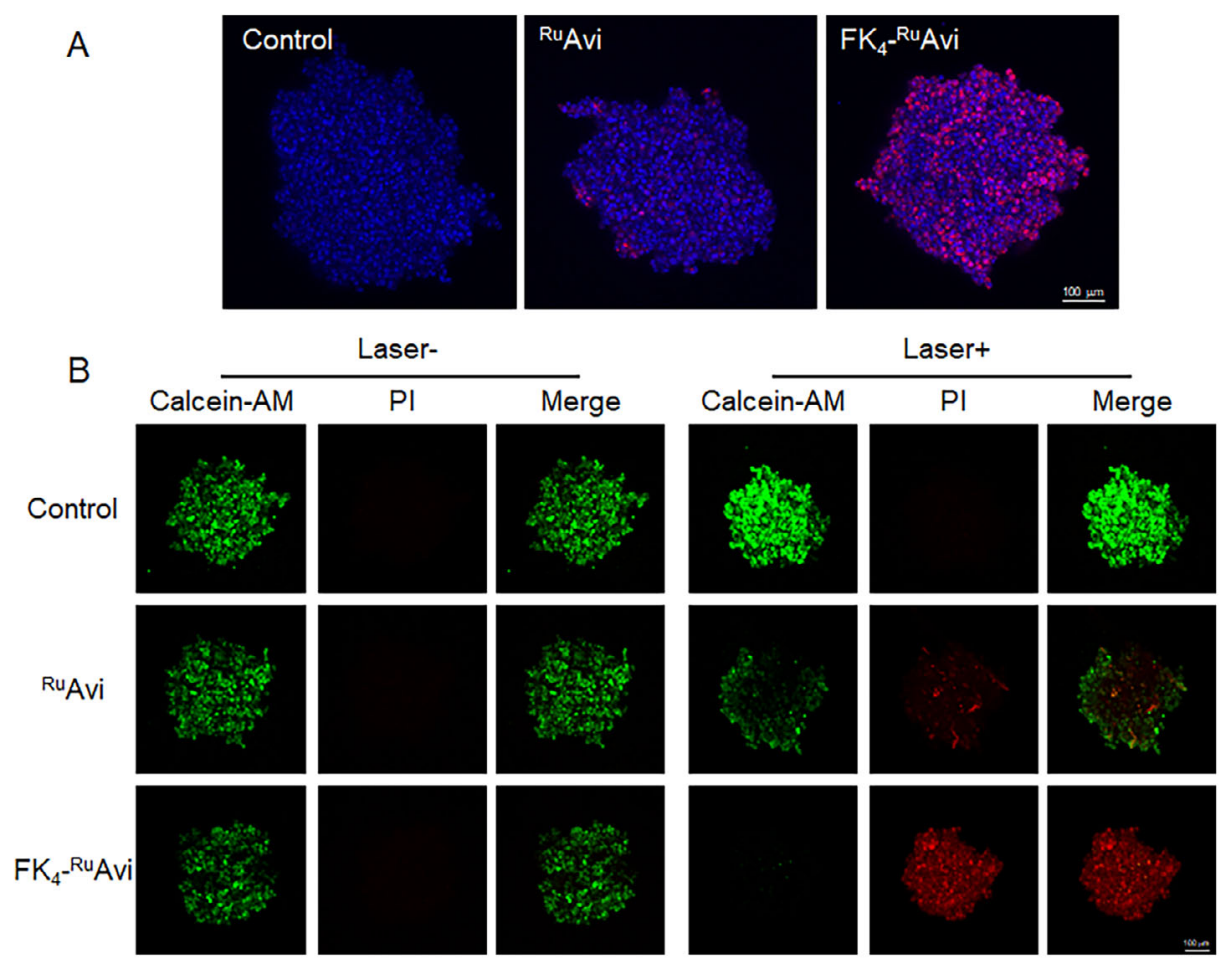
A) Accumulation of 1 µm ^Ru^Avi and 1 µm FK_4_‐^Ru^Avi in the MDA‐MB‐231 spheroids after 48 h treatment (scale bar 100 µm; red channel, Ru‐NH_2_; blue channel, Nucblue). B) Calcein‐AM/PI co‐staining of MDA‐MB‐231 spheroids after treatment with 1 µm ^Ru^Avi and 1 µm FK_4_‐^Ru^Avi for 48 h, with and without laser irradiation (470 nm, 20 mA, 15 min) (scale bar 100 µm; green channel, Calcein‐AM, 2 µm; red channel, PI, 4.5 µm).

Compared to 2D monolayer cells, 3D spheroids are considered to afford a more accurate environment for drug penetration and PS activation, which is better suited for elucidating PDT efficacy.^[^ ^27^ ^]^ Therefore, the MDA‐MB‐231 spheroid was used to evaluate the accumulation of FK_4_‐^Ru^Avi penetration within the spheroids and phototoxicity. The distribution of FK_4_‐^Ru^Avi in MDA‐MB‐231 spheroids was acquired on CLSM, indicating that FK_4_‐^Ru^Avi showed significantly stronger uptake compared to ^Ru^Avi after incubation for 48 h (**Figure** **5**A; Figure S14, Supporting Information). The PDT efficacy of FK_4_‐^Ru^Avi on MDA‐MB‐231 spheroids was then assessed using Calcein‐AM/PI co‐staining (Figure 5B). Notably, FK_4_‐^Ru^Avi showed high toxicity after laser irradiation and no dark toxicity. No significant cell death was observed in the spheroids of the control and ^Ru^Avi‐treated groups, with or without irradiation.

## 3 Conclusion

In conclusion, based on a Mannich‐type reaction we prepared a Ru‐modified Avi platform to obtain ^Ru^Avi by surface modification of tyrosine, with a degree of modification of 57% and preservation of the ^1^O_2_ generation feature of Ru PS. It will be possible to extend this strategy to incorporate other inorganic PSs with NIR wavelength, such as osmium analogs.^[^ ^28^ ^]^ The four biotin‐binding pockets are still accessible after modification to incorporate multiple copies of targeting ligands for enhanced internalization into TNBC in a more controlled fashion compared to other platforms such as human serum albumin.^[^ ^20^ ^]^ The in situ assembly of the ^Ru^Avi platform with functional b‐FK peptides, which targets FPR‐1‐expressing cancer cells, allows rapid screening through this approach. An additive effect with increased cell uptake was observed as the equiv of b‐FK added per avidin tetramer increased from 0 to 4. Furthermore, the optimal FK_4_‐^Ru^Avi transporter showed excellent anticancer effects on FPR‐1 overexpressing MDA‐MB‐231 breast cancer cells both in 2D monolayer and 3D spheroid cell culture, suggesting that it could penetrate effectively and generate ROS to induce cell death upon laser irradiation. Notably, FK_4_‐^Ru^Avi exhibited an IC_50_ value of 0.36 ± 0.08 µm, which is ≈3.5‐fold lower than that of ^Ru^Avi (1.25 ± 0.09 µm), upon irradiation in 2D MDA‐MB‐231 breast cancer cells. We have presented the design of a protein nanocarrier for the targeted delivery of PSs in TNBC, which is in high demand for targeted therapies. This approach has the potential to improve the efficacy of targeted PDT, for example as an adjuvant treatment after breast cancer surgery.

## Conflict of Interest

The authors declare no conflict of interest.

## Supporting information



Supporting Information

## Data Availability

The data that support the findings of this study are available from the corresponding author upon reasonable request.
