# Supplementary material for: A Supramolecular, Triple Negative Breast Cancer‐Targeting Avidin‐Photosensitizer
Source: Macromol Biosci. 2025 Mar 25;25(6):2400610. doi: 10.1002/mabi.202400610 (PMC12169506; doi:10.1002/mabi.202400610)
Supplement: Supplementary file 1 — Supporting Information [file MABI-25-2400610-s001.pdf]

Macromolecular Bioscience

Supporting Information

for *Macromol. Biosci.*, DOI 10.1002/mabi.202400610

A Supramolecular, Triple Negative Breast Cancer-Targeting Avidin-Photosensitizer

Bingjie Gao, Charlotte Schäfers, Seah Ling Kuan and Tanja Weil**

Supporting Information

A Supramolecular, Triple Negative Breast Cancer-targeting Avidin-Photosensitizer

Bingjie Gao[1], Charlotte Schäfers[1,21], Seah Ling Kuan[1,], Tanja Weil[1,]**

B. Gao, C. Schäfers, Dr. S. L. Kuan, Prof. Dr. T. Weil

[1]Department of Synthesis of Macromolecules, Max Planck Institute for Polymer Research, 55128 Mainz, Germany

E-mail: kuan@mpip-mainz.mpg.de; weil@mpip-mainz.mpg.de

[2]Johannes Gutenberg University, Duesbergweg 10-14, 55128 Mainz, Germany

Keywords: supramolecular protein-drug conjugates, photodynamic therapy, targeted delivery, triple-negative breast cancer

General information and materials.

Unless otherwise stated, all chemicals were obtained from commercial sources (Merck, Sigma Aldrich, Thermo Scientific, Fisher Scientific, TCI Chemicals, Broadpharm, and Lumiprobe) and used without further purification. H_2O used for the reactions was obtained from the Millipore purification system. All organic solvents were obtained from Fisher Scientific and used without further purification (HPLC or analytical grades). The peptide cinnamoyl peptide-F(D)LF(D)LFK-NH_2 (FK) was purchased from AmbioPharm with 95% purity (North Augusta, U.S.A). Liquid chromatography-mass spectroscopy (LC-MS) analysis was performed on a Shimadzu LC-MS 2020 equipped with an electrospray ionization source, and an SPD-20A UV-Vis detector. Matrix-Assisted Desorption/Ionization-Time of Flight Mass Spectrometry (MALDI-ToF MS) spectra were acquired on a Bruker Time-of-flight MS rapifleX MALDI ToF-MS equipped with a 10 kHz scanning smartbeam 3D laser (Nd: YAG at 355 nm) and a 10-bit 5 GHz digitizer. Vivaspin 500 and Vivaspin 6 (MWCO 10 kDa) from Sartorius (Sartorius Lab Instruments GmbH & Co. KG, Goettingen, Germany) were used, with either the Fresco 21 microcentrifuge or the Eppendorf centrifuge 5810R, for ultrafiltration. UV-visible measurements were performed using a Nanodrop 2000 spectrophotometer.

Synthesis of Ru-NH_2.

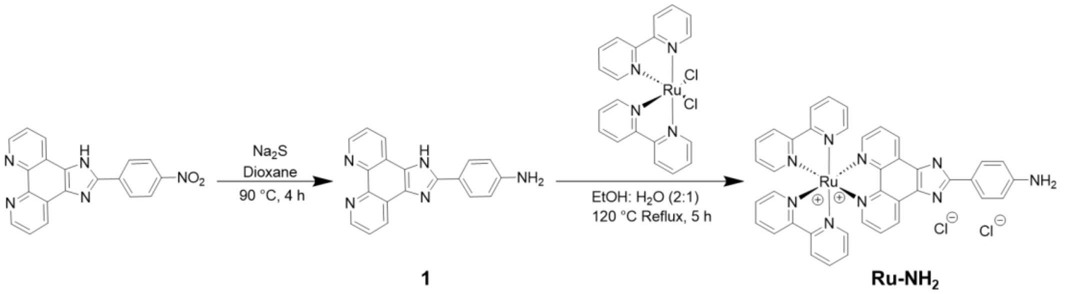

Scheme S1: Reaction scheme for the synthesis of Ru-NH_2 adapted from the published procedure.

The synthesis of Ru-NH_2 was adapted from the reported protocol with minor changes.[1] A suspension of 2-(4-nitrophenyl)-1H-imidazo[4,5-f][1,10]-phenanthroline (413.6 mg, 1.2 mmol, 1 equiv) was prepared in 1,4-dioxane (20 mL) and heated to 90 °C. Separately, sodium sulfide

(Na_2S, 945.6 mg, 12.1 mmol, 10 equiv) was dissolved in water and heated to 90 °C. The warm Na_2S solution was then added to the yellow suspension, and the mixture was refluxed at 90 °C for 4 h. After cooling, the dioxane was removed by rotary evaporation, leading to the formation of orange-colored precipitates. The solid was filtered, washed with water followed by diethyl ether (Et_2O), and dried to yield an ochre-colored solid as the compound **1,** 4-(1H-imidazo[4,5-f][1,10]phenanthrolin-2-yl)aniline.

Subsequently, compound 1 (313.2 mg, 1.0 mmol, 1 equiv) and cis-Bis(2,2′-bipyridine)dichlororuthenium(II) hydrate (535.9 mg, 1.1 mmol, 1.1 equiv) were dissolved in a 15 mL ethanol-water mixture (2:1, ethanol) in a 25 mL round-bottom flask. The mixture was fitted with a condenser and refluxed at 120 °C for 5 h, during which the solution's color changed from dark violet to red. After cooling to room temperature, the solvent was removed by rotary evaporation. The residue was dissolved in ethanol, and Et_2O was added, precipitating red crystals. The red crystals were filtered and further purified by HPLC with the mobile phase starting from 100% solvent A (0.1% TFA in water) and 0% solvent B (0.1% TFA in acetonitrile) with a flow rate of 25 mL/min, rising to 100% solvent B in 23 min. It remained in this state for 3 min. Solvent B concentration was then finally lowered to 0% over 5 min. Absorbance was monitored at 470 and 254 nm. 626.2 mg (0.9 mmol, 86%) of the product was obtained after lyophilization; ^{1}H NMR (400 MHz, MeOD-*d*4) δ = 9.12 (dd, *J*=8.4, 1.0, 2H), 8.78 – 8.67 (m, 4H), 8.17 (td, *J*=7.9, 1.5, 2H), 8.13 – 8.01 (m, 6H), 7.94 (ddd, *J*=5.6, 1.5, 0.7, 2H), 7.86 (ddd, *J*=8.4, 5.3, 0.9, 2H), 7.70 – 7.63 (m, 2H), 7.54 (ddd, *J*=7.6, 5.6, 1.3, 2H), 7.31 (ddd, *J*=7.2, 5.7, 1.3, 2H), 6.97 – 6.88 (m, 2H). This is in agreement with the literature report.[2]

LC-MS: T_R: 3.50 min, *m/z*: 724.2 [M-H-2Cl]$^+$ (calculated mass: 725.2, formula: $C_{39}H_{29}N_9Ru$)

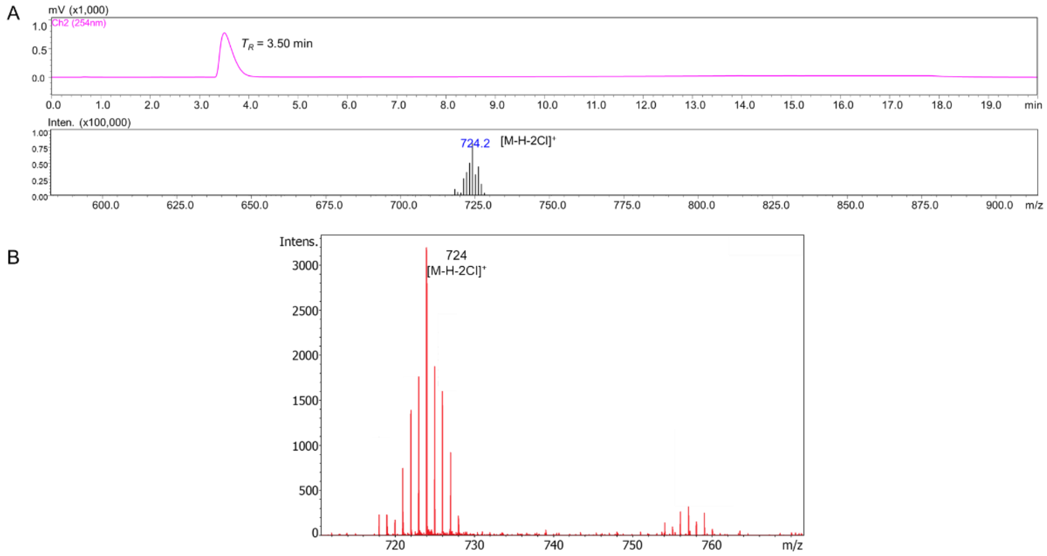

Figure S1. (A) LC-MS spectrum of Ru-NH_2 with LC trace at 254 nm, T_R: 3.50 min, positive ionization mode; *m/z* found: $[M-H-2Cl]^+$ = 724.2 g/mol (calculated mass: 725.2 formula: $C_{39}H_{29}N_9Ru$). (B) MALDI-TOF MS characterization of Ru-NH_2 *m/z* found: $[M-H-2Cl]^+$ = 724 g/mol using Cyano-4-hydroxycinnamic acid (CHCA) as the matrix,

Ru-NH_2 modification of Avidin.

In a 25 mL round-bottom flask, 10 mg Avidin (151.5 nmol, 1 equiv) was dissolved in 10 mL PBS (pH 5.0), followed by the addition of formaldehyde (37 wt. %, 50 μL) and Ru-NH_2 (7.58 μmol, 50 equiv, 5.49 mg). The mixture was stirred gently at 37 °C for 72 h in the dark and washed several times with MilliQ through Vivaspin 6 (MWCO 10kDa) ultrafiltration to remove excess Ru-NH_2, then lyophilized to afford powder for further use.

ÄKTA pure™ protein purification system.

Size exclusion chromatography (SEC) was performed using the ÄKTA pure ™ protein purification system (Cytiva Life Sciences, Marlborough, U.S.A). Separation was achieved using a Superdex™ 75 Increase (Cytiva Life Sciences, Marlborough, U.S.A) column with 50 mM PB (pH 7.4) as the mobile phase. A 500 µL injection loop was used to introduce 500 µL of a 1 mg/mL ^{Ru}Avi and Avi sample into the system, and elution was carried out at a flow rate of 0.5 mL/min. The total collected volume was 1.5 times the column volume. Absorbance was monitored at 280 nm to detect the presence of proteins and at 470 nm to identify Ru-labeled

molecules.

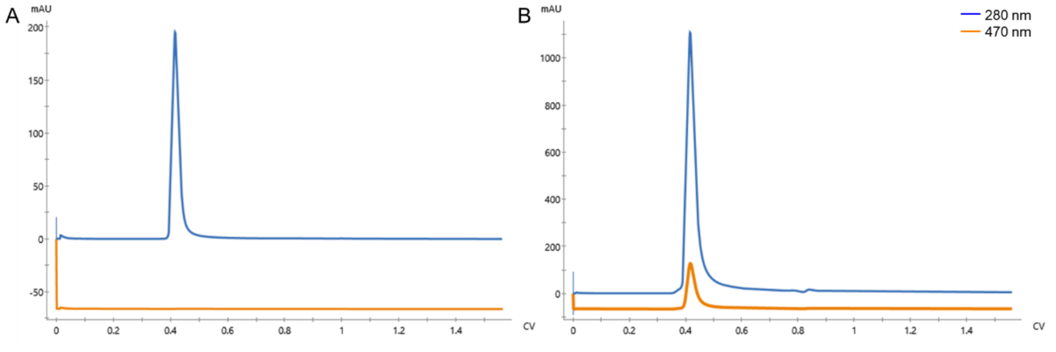

Figure S2. Size exclusion chromatography of tetrameric (A) Avi and (B) ^{Ru}Avi with the absorbance monitored at 280 nm (blue) and 470 nm (orange).

HiTrap SPFF. Cation exchange chromatography was performed using the ÄKTA pure™ protein purification system (Cytiva Life Sciences, Marlborough, U.S.A). The separation was carried out using a HiTrap SPFF (Cytiva Life Sciences, Marlborough, U.S.A) column, with 50 mM PB (pH 6.5) containing 1 M NaCl as the mobile phase. A flow rate of 1 mL/min was maintained throughout the run. The elution process was monitored by measuring absorbance at 280 nm to detect proteins and at 470 nm to identify Ru-labeled molecules.

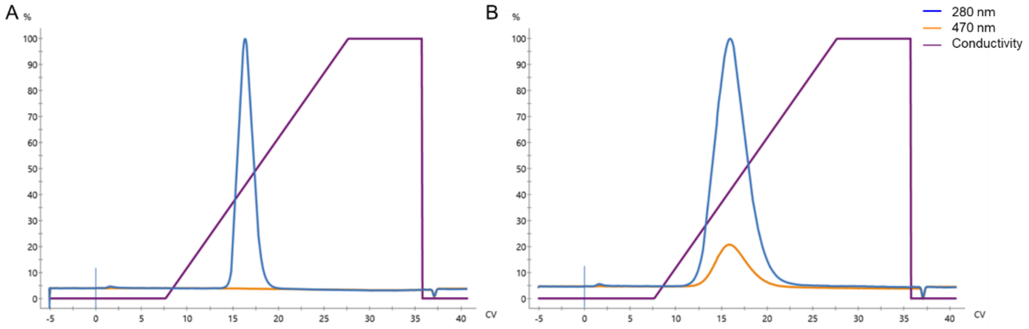

Figure S3. Cation exchange chromatography of tetrameric (A) Avi and (B) ^{Ru}Avi with the absorbance monitored at 280 nm (blue) and 470 nm (orange).

Ru-NH$_2$ quantification.

To determine the degree of Ru-NH$_2$ modification in ^{Ru}Avi, the Ru-NH$_2$ standard solutions ranging from 0.001 mg/mL to 1 mg/mL were used to generate a standard curve with absorption

at 470 nm. Subsequently, the absorption of a 1 mg/mL ^{Ru}Avi solution was also measured at 470 nm, yielding a Ru-NH_2 labeling efficiency of 57%. All measurements were obtained in triplicate.

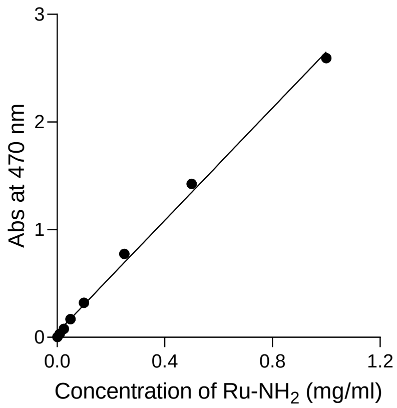

Figure S4. The working curve for quantifying Ru-NH_2 labeling on ^{Ru}Avi with Ru-NH_2 solution as standards.

<u>Singlet oxygen ($^1O_2$) generation measurements.</u>

The capacity of ^{Ru}Avi to generate singlet oxygen ($^1O_2$) in an aqueous solution under 470 nm irradiation was assessed using 9,10-anthracenediyl-bis(methylene)dipropanedioic acid (ABDA) as a probe. ABDA was first solubilized in DMSO at a concentration of 10 mM, and subsequently mixed with solutions of 0.2 mg/mL Ru-NH_2 and ^{Ru}Avi in 50 mM PB buffer (pH 7.4) at a volume ratio of 1:4. The absorbance intensity and spectra of ABDA solutions were recorded after 10 min of irradiation.

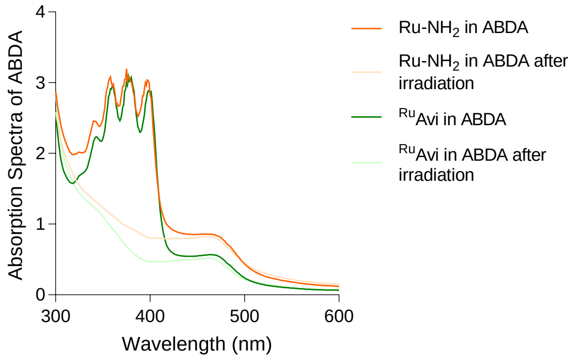

Figure S5. Absorption spectra of ABDA mixtures with Ru-NH_2 or ^{Ru}Avi before and after 470 nm laser arrays irradiation (20 mA, 10 min).

MALDI-ToF MS characterization.

The modified protein was characterized using MALDI-ToF MS. The MS data is plotted in Origin and processed as follows: Smoothing was performed using the Savitzky-Golay method with a 20-point window and polynomial order of 3, while other parameters remain at default. The smoothed data is normalized to a maximum value of 100%, and a final line plot of the normalized data is generated.

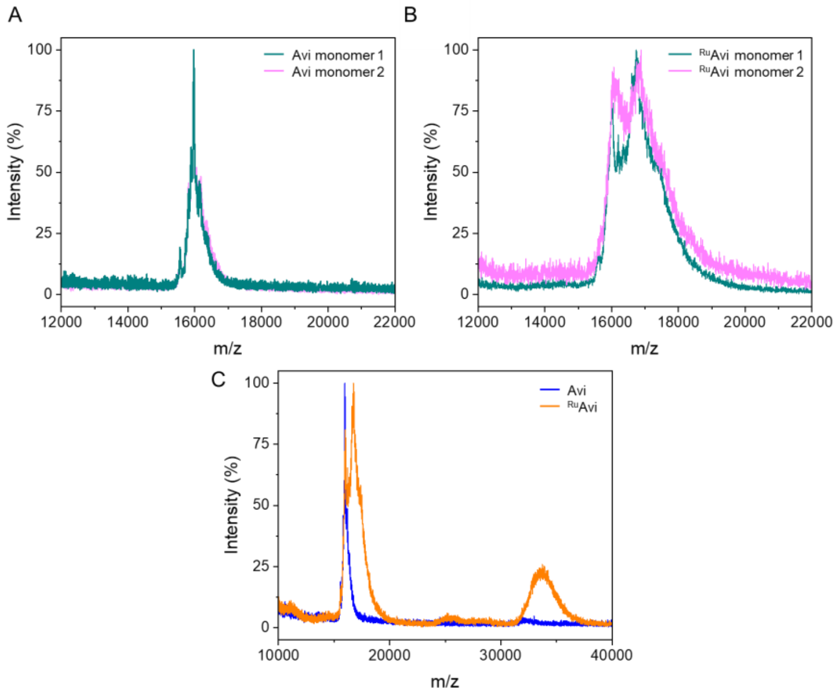

Figure S6. Representative MALDI-ToF MS characterization of monomeric (A) Avi *m/z* = 15971 ± 3 Da, and (B) ^{Ru}Avi *m/z* = 16757 ± 24 Da from two batches with sinapinic acid (SA) as the matrix. (C) MALDI-ToF MS of Avi and ^{Ru}Avi from 10−40000 Da. The peak at 33514 Da corresponds to the dimeric protein.

Stoichiometry of ^{Ru}Avi with biotin-PEG$_3$-Cyanine5 (b-Cy5).

To determine the stoichiometric ratio of biotinylated molecules required to saturate the biotin-binding pockets of ^{Ru}Avi, biotin-PEG$_3$-Cyanine5 (b-Cy5) was used. In brief, 0 to 6 equiv of 1 mg/ml b-Cy5 were added to 100 µL of a 1 mg/mL ^{Ru}Avi (1.5 nmol, 1 equiv) and Avi (1.5 nmol, 1 equiv). The mixture was gently agitated at room temperature for 30 min, followed by ultrafiltration with Vivaspin 500 (MWCO 10 kDa) to remove the excess b-Cy5. Triplicates of

each sample (25 µL) were then transferred into a clear, flat-bottom 384-well plate (UV-star®, Greiner Bio-one GmbH, Frickenhausen, Germany). UV-Vis absorption at 280 nm, 470 nm, and 651 nm was measured using a Tecan Spark 20M microplate reader (Tecan Trading AG, Männedorf, Switzerland). A plot of the absorbance ratio at 651 nm to 280 nm was generated to determine the stoichiometric ratio required for full saturation of the binding pockets.

Assembly of the transporter FK_n-^{Ru}Avi (n = 1–4, corresponding to the number of equiv. of b-FK added per tetrameric Avi).

Typically, in 900 µL of 50 mM PB (pH 7.4), 1 mg of ^{Ru}Avi (10 mg/mL in MilliQ water, 15 nmol, 1 equiv.) was added. Then, 14 µL of 5 mg/mL b-FK (70 µg, 60 nmol, 4 equiv) was introduced to the ^{Ru}Avi solution. The mixture was vortexed and shaken at room temperature for 30 min. The purification was carried out using Vivaspin 500 (MWCO 10 kDa) and centrifugation with 3x 500 µL of 50 mM PB (pH 7.4). The concentration of the transporter solution was determined by measuring absorbance at 470 nm using a Nanodrop, and the FK_4-^{Ru}Avi solution was stored at 4°C in the dark for future use.

For FK_1-^{Ru}Avi, FK_2-^{Ru}Avi, and FK_3-^{Ru}Avi, the equiv of b-FK were adjusted to 1, 2, and 3 accordingly, while all other conditions remained the same.

Dynamic light scattering (DLS) of the transporter FK_4-^{Ru}Avi.

Similarly, the stock solutions of Avi, ^{Ru}Avi, and FK_4-^{Ru}Avi were diluted to 1 mg/mL using 10 mM PBS (pH 7.4). Light scattering measurements were performed on a Zetasizer Nano S90 (Malvern Panalytical Ltd, UK), using a helium-neon laser with a wavelength of 633 nm as the light source. For each measurement, 200 µL of the sample was added to the sample cell, and calibration was carried out at 25 °C for 60 seconds. The results recorded at the default measurement angle were averaged over three sets of 30 scans each, with the relationship between volume and hydrodynamic size documented.

Zeta potential measurements of the transporter FK_4-^{Ru}Avi.

The stock solutions of Avi, ^{Ru}Avi, and FK_4-^{Ru}Avi were diluted to 0.2 mg/mL using 1 mM KCl solution. Zeta potential measurements were conducted using the Zetasizer Nano Z (Malvern

Panalytical Ltd, UK). A volume of 800 μL of each sample was added to the sample cell, ensuring the removal of air bubbles. After a 60-second calibration at 25°C, the measurements were performed. The results were reported as the average of three sets of 30 scans each.

Table S1. Zeta potential values of Avi, ^{Ru}Avi, and FK$_4$-^{Ru}Avi in 1 mM KCl.

	Zeta Potential Value (mV)
Avi	2.5±0.6
^{Ru}Avi	20.6±1.6
FK$_4$-^{Ru}Avi	15.3±0.4

Sodium dodecyl sulfate polyacrylamide gel electrophoresis (SDS-PAGE) analysis.

To conduct SDS-PAGE analysis under denatured and non-denatured conditions, a 10% separation gel and a 6% stacking gel were prepared using a bis-Tris buffer system. To assess the stability of the assembled FK$_4$-^{Ru}Avi complex, samples were stored at 4°C for one month following assembly. For denatured samples, 5.8 μL of 50 mM PB buffer (pH 7.4), 2 μL of a 1 mg/mL protein sample, 1.2 μL of 1 M dithiothreitol (DTT), and 3 μL of NuPAGE™ LDS Sample Buffer (4X, Thermo Fisher Scientific Inc., Waltham, USA) were combined. This mixture was heated at 95°C for 10 min and then allowed to cool. For non-denatured samples, 7.0 μL of 50 mM PB, 2 μL of the protein sample, and 3 μL of the same sample buffer were mixed without heating. 10 μL of each sample was loaded into the gel, which was run at 120 V through the stacking gel and then increased to 150 V through the separation gel. The gel was initially scanned under the fluorescence channel, followed by staining with Coomassie Blue to visualize the proteins. Results were documented using a Gel Doc Gel Documentation System (Bio-Rad Laboratories GmbH, Hercules, USA).

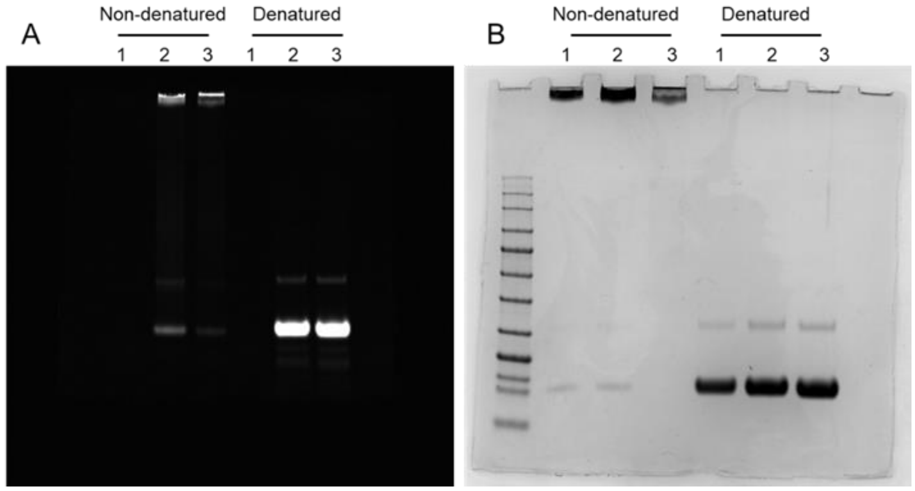

Figure S7. Sodium dodecyl sulfate polyacrylamide gel electrophoresis (SDS-PAGE) of (1) Avi, (2) ^{Ru}Avi, and (3) FK_4-^{Ru}Avi showing the stability over one month scanned (A) fluorescence detection and (B) Coomassie staining.

2D Cell culture

MDA-MB-231 cells were cultured in T75 tissue culture flasks (Greiner bio-one, Frickenhausen, Germany) at 37 °C and 5% CO_2 in Dulbecco's modified Eagle medium (DMEM, Gibco, Thermo Fisher Scientific Inc., Waltham, USA), supplemented with 10% fetal bovine serum (FBS, Gibco, Thermo Fisher Scientific Inc., Waltham, USA) and 1% penicillin-streptomycin (10.000 U/mL, Gibco, Thermo Fisher Scientific Inc., Waltham, USA). Cells were routinely washed with Dulbecco's phosphate-buffered saline (DPBS), trypsinized (TrypLE® Express, Gibco, Thermo Fisher Scientific Inc., Waltham, USA), and reseeded two to three times per week.

Cellular internalization of the transporter FK_n-^{Ru}Avi.

MDA-MB-231 cells were seeded into an 8-well chambered coverslip (ibidi GmbH, Gräfelfing, Germany) at a density of 20,000 cells per well and incubated overnight to allow for attachment. Following incubation, the cells were treated with 200 µL of 1 µM ^{Ru}Avi, along with assembled FK_1-^{Ru}Avi, FK_2-^{Ru}Avi, FK_3-^{Ru}Avi, and FK_4-^{Ru}Avi, and incubated for 24 h. After treatment, the samples were removed, and the cells were rinsed three times with DPBS, fixed with 4% paraformaldehyde (Carl Roth GmbH + Co. KG, Karlsruhe, Germany), and stained with NucBlue (Thermo Fisher Scientific Inc., Waltham, USA). Confocal Laser Scanning Microscopy was performed using a STELLARIS 8 Leica DMi8 microscope (Leica Microsystems, Wetzlar,

Germany) with a 40x oil immersion objective.

To confirm the temperature-dependent uptake of the transporter FK$_4$-^{Ru}Avi, cells were pre-cooled at 4°C for 30 min, then treated with 200 µL of 1 µM ^{Ru}Avi and FK$_4$-^{Ru}Avi. The plate was kept at 4°C for 4 h, while a control plate was incubated under standard conditions. Both plates then underwent the standard rinsing, fixation, and NucBlue staining procedures. During the confocal measurements, the same gain and intensity were set for all images. Images in the Ru channel were first converted to 8-bit format and then adjusted to a uniform threshold range of 20-255. Quantification of mean fluorescence intensity was conducted using ImageJ software. The specific steps in ImageJ included selecting 'Analyze', followed by 'Set Measurements', and checking options for 'Area', 'Integrated Density', and 'Mean Gray Value'. Measurements were then taken from three independent images.[3]

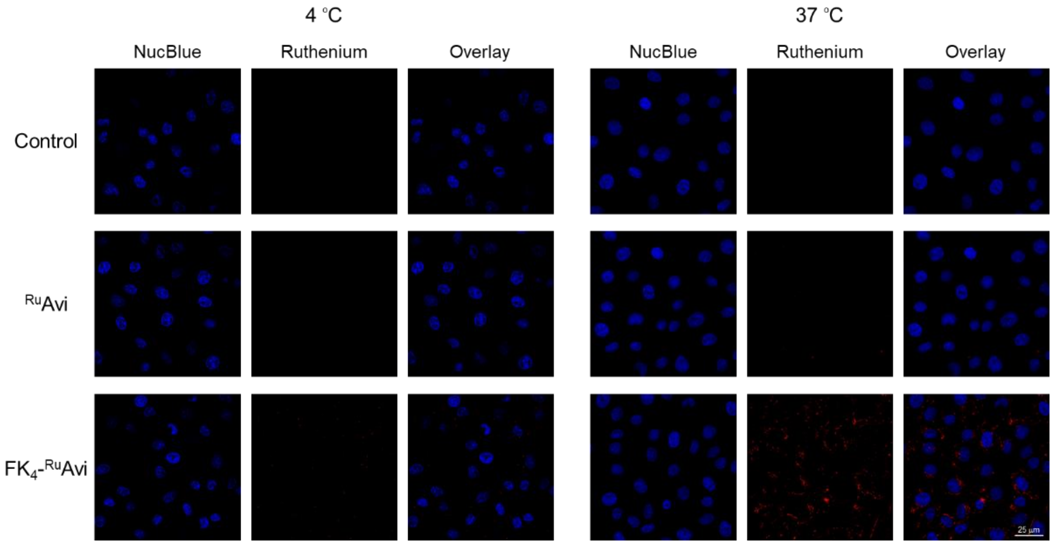

Figure S8. Separate channels and overlay of confocal laser scanning micrographs in **Figure 3C** (scale bar 25 µm).

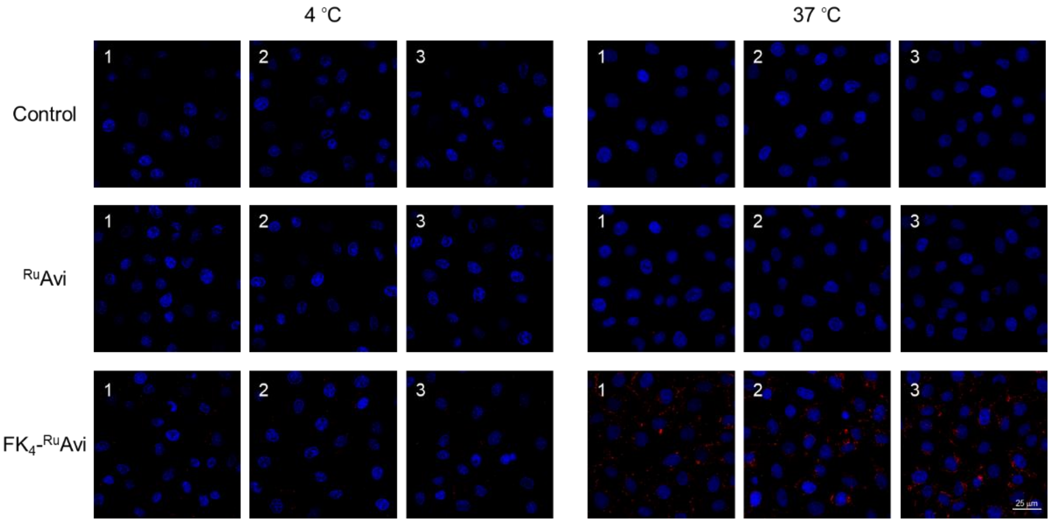

Figure S9. Images captured at 4°C and 37°C, quantitatively analyzed using ImageJ to measure the mean fluorescence intensity in the Ru channel. Analysis was conducted on three independent images ($22 \leq n \leq 35$ cells per image; scale bar 25 μm; red channel, Ru-NH_2; blue channel, Nucblue).

To saturate the FPR-1 receptor on the MDA-MB-231 cell surface, cells were treated with concentrations of 10 μM and 50 μM b-FK. Subsequently, they were incubated with 200 μL of 1 μM FK_4-^{Ru}Avi for 4 hours. Following incubation, standard procedures of rinsing, fixation, and NucBlue staining were performed. Consistent settings were maintained during confocal measurements. Images in the Ru channel were converted to an 8-bit format, followed by adjustment to a consistent threshold range of 20-255. The mean fluorescence intensity was quantified using ImageJ by selecting 'Analyze', then 'Set Measurements', and enabling the options for 'Area', 'Integrated Density', and 'Mean Gray Value'. Measurements were subsequently carried out on three independent images.

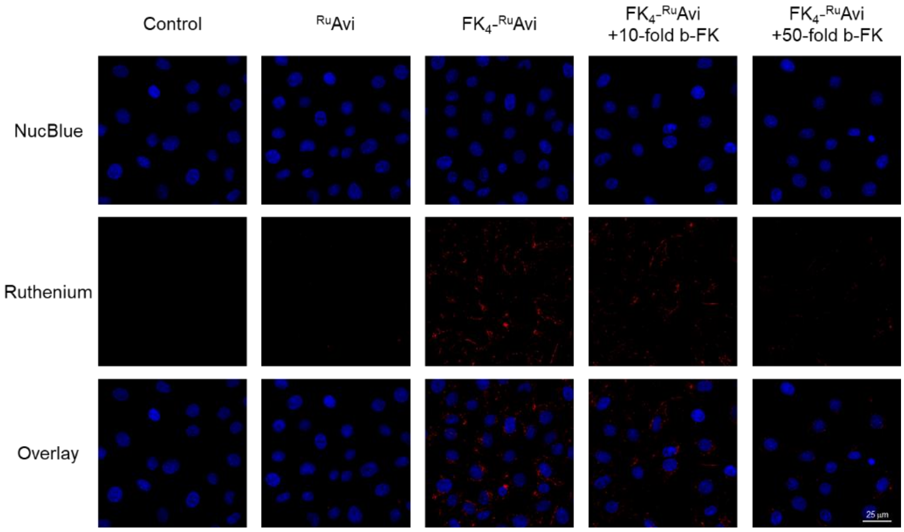

Figure S10. Separate channels and overlay of confocal laser scanning micrographs showing the intracellular internalization of FK_4-^{Ru}Avi with excess b-FK at 37°C for 4 hours. These images illustrated how blocking the FPR-1 receptor reduced the internalization of FK_4-^{Ru}Avi. (scale bar 25 μm).

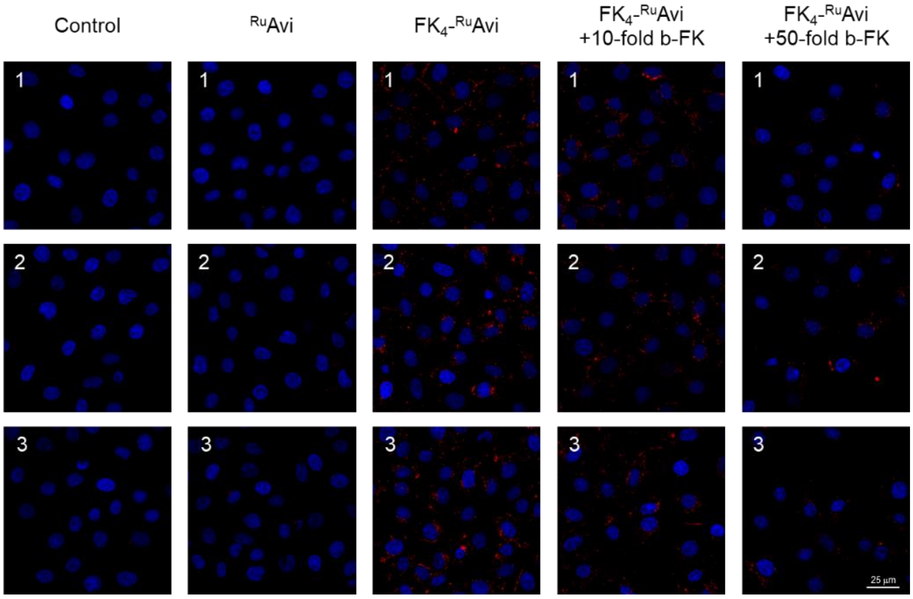

Figure S11. Images obtained following treatment with excess b-FK, quantitatively analyzed using ImageJ to measure the mean fluorescence intensity in the Ru channel. Analysis was conducted on three independent images ($15 \leq n \leq 35$ cells per image; scale bar 25 μm; red channel, Ru-NH_2; blue channel, Nucblue).

Cellular internalization quantification with cell lysis.

MDA-MB-231 cells were seeded at a density of 5000 cells per well in a 96-well plate (Corning Inc., New York, USA) and allowed to adhere overnight. Cells were then treated with 1 μM of ^{Ru}Avi, FK$_1$-^{Ru}Avi, FK$_2$-^{Ru}Avi, FK$_3$-^{Ru}Avi, and FK$_4$-^{Ru}Avi and incubated for 48 h. After incubation, cells were washed three times with cold DPBS and lysed using 100 μL of RIPA buffer (SERVA Electrophoresis GmbH, Heidelberg, Germany) per well. Then the fluorescence intensity of lysates was measured using a Tecan Spark 20M microplate reader (Tecan Trading AG, Männedorf, Switzerland) with an excitation wavelength of 470 nm and an emission wavelength of 640 nm. The fluorescence intensity of each sample was recorded, and the results were averaged from five wells per condition. Statistical analysis was performed using one-way ANOVA to assess the significance of differences in cellular uptake between the samples, $*p < 0.05$, $**p < 0.01$, $***p < 0.001$, and $****p < 0.0001$.

ROS Staining

Intracellular ROS generation was monitored using the 2',7'-dichlorodihydrofluorescein diacetate (DCFH-DA) probe (Thermo Fisher Scientific Inc., Waltham, USA). MDA-MB-231 cells were seeded at a density of 30,000 cells per well into a 24-well plate (Corning Inc., New York, USA) with 500 μL of complete medium and allowed to adhere overnight in the incubator. Following this, the cells were treated with 500 μL of 2 μM Ru-NH_2, 0.5 μM ^{Ru}Avi, and 0.5 μM FK$_4$-^{Ru}Avi, and incubated in the dark for 24 h. After incubation, the cells were rinsed with DPBS and incubated with 500 μL of 10 μM DCFH-DA for 30 min. The laser-treated plates were then exposed to irradiation using a lamp (470 nm, 15 mA, 5 min), while the control plates were kept in the dark. Images were captured using the All-in-One Fluorescence Microscope BZ-X800 system (Keyence Corporation, Osaka, Japan) with a 20x air objective (Filter cube, GFP, Ex 470/40, Em 525/50).

Cell Viability Assay

Phototoxicity and dark toxicity were assessed using an MTT assay (3-[4,5-dimethylthiazol-2-yl]-2,5-diphenyl tetrazolium bromide, TCI Deutschland GmbH, Eschborn, Germany). MDA-MB-231 cells were seeded at a density of 5000 cells per well in a 96-well plate (Corning Inc., New

York, USA) with 100 μL of complete medium and allowed to adhere overnight in the incubator. Following this, the cells were treated with $Ru-NH_2$, ^{Ru}Avi, and FK_4-^{Ru}Avi ranging from 2.3 nM to 2.3 μM (in terms of the concentration of $Ru-NH_2$), and incubated in the dark for 48 h. At 11.5 μM, we observed dark toxicity of FK_4-^{Ru}Avi (Figure S7), which could be induced due to precipitation in the cell culture. After incubation, cells were rinsed with DPBS, a fresh complete medium was added, and the cells were irradiated with or without a lamp (470 nm, 15 mA, 5 min). After an additional 24 h of incubation, the medium was replaced with 100 μL of MTT working solution (0.5 mg/mL in 90% DMEM) per well, and the cells were incubated for 4 h. The medium was then carefully removed, and 100 μL of DMSO was added to each well to dissolve the formazan crystals formed in the living cells. The optical density of each well was measured at 490 nm using a Tecan Spark 20M microplate reader (Tecan Trading AG, Männedorf, Switzerland). Samples were measured with three independent experiments, with each experiment conducted in quintuplicates.

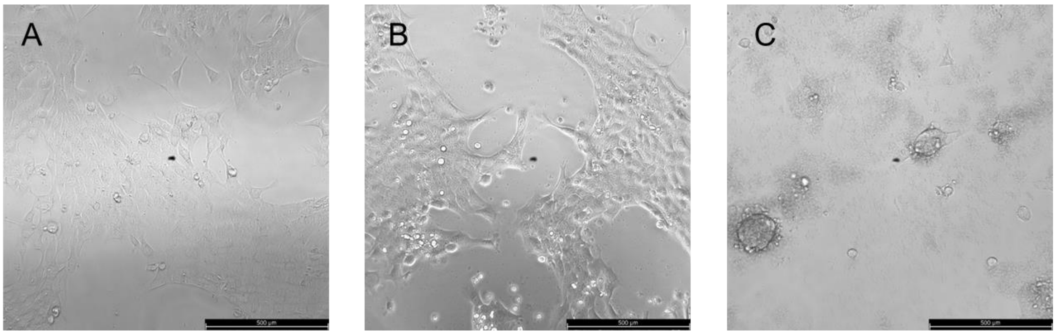

Figure S12. Representative bright-field images of MDA-MB-231 cells treated with (A) control, (B) 11.5 μM ^{Ru}Avi, and (C) 11.5 μM FK_4-^{Ru}Avi (based on Ru concentration), illustrating distinct outcomes. (Scale bar, 500 μm) Under dark conditions, cells treated with 11.5 μM ^{Ru}Avi appeared healthy and maintained normal morphology, while those treated with 11.5 μM FK_4-^{Ru}Avi exhibited signs of cell death. This may be attributed to the precipitation of FK_4-^{Ru}Avi at concentrations higher than 11.5 μM in the cell culture environment.

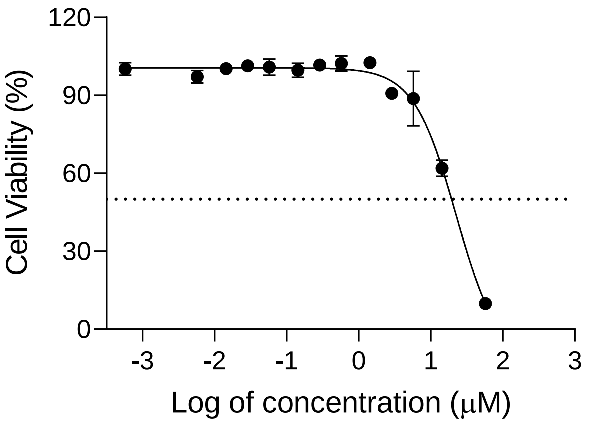

Figure S13. Cell viability of b-FK against MDA-MB-231 cells with treatment for 48 h, yielding an IC_{50} value of 42.30 ± 4.43 μM (quintuplicates in two independent experiments), showing that b-FK was not toxic within the concentration range tested for FK_4-^{Ru}Avi.

Table S2. The IC_{50} value of Ru-NH_2, ^{Ru}Avi, and FK_4-^{Ru}Avi without and with 470 nm laser irradiation (quintuplicates in three independent experiments).

	Laser (-) IC_{50} μM	Laser (+) IC_{50} μM
Ru-NH_2	> 2.30	0.58 ± 0.01
^{Ru}Avi	> 2.30	1.25 ± 0.09
FK_4-^{Ru}Avi	> 2.30	0.36 ± 0.08

Live-Dead Staining

MDA-MB-231 cells were seeded at a density of 5000 cells per well into a 96-well plate (Corning Inc., New York, USA) with 100 µL of complete medium and allowed to adhere overnight in the incubator. The next day, cells were treated with 100 µL of 2 µM Ru-NH_2, 0.5 µM ^{Ru}Avi, and 0.5 µM FK_4-^{Ru}Avi, followed by incubation in the dark for 48 h. After treatment, cells were rinsed with DPBS, a fresh complete medium was added, and the cells were exposed to light irradiation (470 nm, 15 mA, 5 min) or kept in the dark. After an additional 24 h of incubation, the cells were co-stained with 100 µL of 2 µM Calcein-AM (Thermo Fisher Scientific Inc., Waltham, USA) and

4.5 µM Propidium Iodide (Thermo Fisher Scientific Inc., Waltham, USA) for 30 min to distinguish live cells from dead cells. Images were captured using an All-in-One Fluorescence Microscope BZ-X800 system (Keyence Corporation, Osaka, Japan) with a 20x air objective, employing filter cubes for Calcein-AM (GFP, Ex 470/40, Em 525/50) and Propidium Iodide (TRITC, Ex 545/25, Em 605/70).

3D Cell culture

To generate MDA-MB-231 spheroids, MDA-MB-231 cells were seeded with a density of 10000 cells per well in 100 µL complete medium in ultra-low attachment 96-well plates (4520, Corning Inc., New York, USA), and then centrifuged at 1200 rpm for 5 min. Cells were kept in an incubator for a week to form the spheroids.

3D Cellular internalization

MDA-MB-231 spheroids were rinsed with DPBS and then incubated with 100 µL of 1 µM ^{Ru}Avi, and 1 µM FK_4-^{Ru}Avi for 48 h. Then, samples were removed and spheroids were rinsed with DPBS, fixed with 4% paraformaldehyde solution (Carl Roth GmbH + Co. KG, Karlsruhe, Germany), and stained with Nucblue reagent containing 0.1% Triton X-100 (Thermo Fisher Scientific Inc., Waltham, USA). After transferring spheroids to an 8-well plate (ibidi GmbH., Gräfelfing, Germany), Confocal Laser Scanning Microscopy was performed on a STELLARIS 8 Leica DMi8 microscope (Leica Microsystems, Wetzlar, Germany) with a 20x air objective.

For semi-quantitative analysis, three independent spheroids were measured. The quantification of 3D accumulation using ImageJ was obtained first by the summed intensity (integral) of the Ru channels for each spheroid. A method similar to the 2D analysis was then employed. The images were initially converted to an 8-bit format, and a consistent threshold range of 20-255 was established for all images. Following this, the "Analyze > Set Measurements" option was set up to abstract Area, Integrated Density, and Mean Gray Value. For mean fluorescence intensity analysis, three tumor spheroids per sample were selected to assess the 3D accumulation within the tumor spheroids.

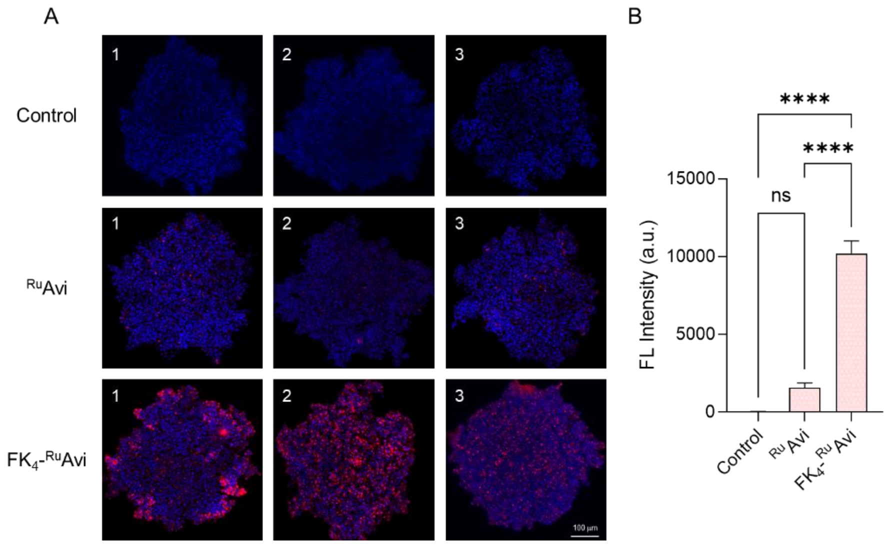

Figure S14. (A) Images and (B) fluorescence intensity quantification for the accumulation of 1 μM ^{Ru}Avi and 1 μM FK$_4$-^{Ru}Avi in the MDA-MB-231 spheroids after 48 h treatment, quantitatively analyzed using ImageJ to measure the mean fluorescence intensity in the Ru channel (scale bar 100 μm; red channel, Ru-NH$_2$; blue channel, Nucblue). Statistical analysis was performed using one-way ANOVA to assess the significance of differences in cellular uptake between the samples, *$p < 0.05$, **$p < 0.01$, ***$p < 0.001$, and ****$p < 0.0001$. The data is shown as mean ± SEM, n = 3.

3D Live-Dead Staining

MDA-MB-231 spheroids were rinsed with DPBS and then incubated with 100 μL of 1 μM ^{Ru}Avi, and 1 μM FK$_4$-^{Ru}Avi for 48 h. After removing the samples and replacing them with a fresh complete medium, spheroids were exposed with or without irradiation (470 nm, 20 mA, 5 min). After 24 h of further incubation, spheroids were co-stained with 100 μL of 2 μM Calcein-AM (Thermo Fisher Scientific Inc., Waltham, USA) and 4.5 μM Propidium Iodide (Thermo Fisher Scientific Inc., Waltham, USA) for 45 min. The images were obtained on a STELLARIS 8 Leica DMi8 microscope (Leica Microsystems, Wetzlar, Germany) with a 20x air objective.

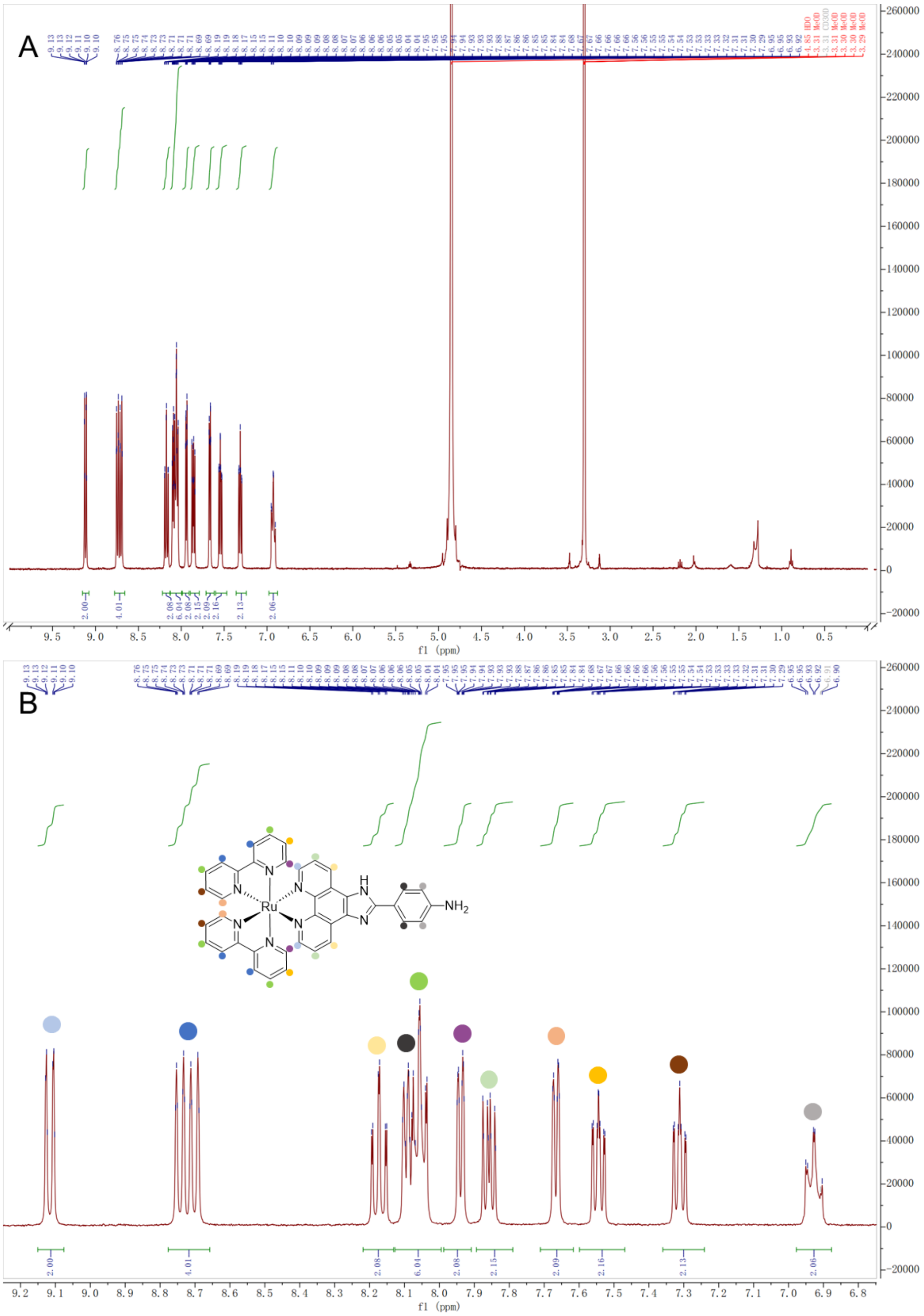

Figure S15. (A) Full and (B) expanded ^{1}H-NMR (400 MHz, MeOD-*d*4) spectra of Ru-NH_2.

References.

[1] S. Chakrabortty, B. K. Agrawalla, A. Stumper, N. M. Vegi, S. Fischer, C. Reichardt, M. Kögler, B. Dietzek, M. Feuring-Buske, C. Buske, S. Rau, T. Weil, *J. Am. Chem. Soc.* **2017**, *139*, 2512.

[2] A. Stumper, M. Lämmle, A. K. Mengele, D. Sorsche, S. Rau, *Eur. J. Inorg. Chem.* **2018**, *2018*, 586.

[3] V. Mehta, K.-L. Pang, D. Rozbesky, K. Nather, A. Keen, D. Lachowski, Y. Kong, D. Karia, M. Ameismeier, J. Huang, Y. Fang, A. del Rio Hernandez, J. S. Reader, E. Y. Jones, E. Tzima, *Nature* **2020**, *578*, 290.

Author Contribution

Bingjie Gao: Initiating the project and conducting experimental work, data analysis, and processing, preparing figures, and drafting the manuscript.

Charlotte Schäfers: Performing protein modification and optimization of experimental conditions.

Seah Ling Kuan: Discussion of the design, concept, and results, correcting the manuscript, and supervision of Bingjie Gao.

Tanja Weil: Involved in the inception and design concept, discussion of the concept and results, correcting the manuscript, acquiring funding for the project, and supervision of Bingjie Gao.
